# Lipidomic studies reveal two specific circulating phosphatidylcholines as surrogate biomarkers of the omega-3 index

**DOI:** 10.1016/j.jlr.2023.100445

**Published:** 2023-09-18

**Authors:** Ritchie Ly, Brittany C. MacIntyre, Stuart M. Philips, Chris McGlory, David M. Mutch, Philip Britz-McKibbin

**Affiliations:** 1 Department of Chemistry and Chemical Biology, McMaster University, Hamilton, ON, Canada; 2 Department of Human Health and Nutritional Sciences, University of Guelph, Guelph, ON, Canada; 3 Department of Kinesiology, McMaster University, Hamilton, ON, Canada; 4 School of Kinesiology and Health Studies, Queen’s University, Kingston, ON, Canada

**Keywords:** Biomarkers, Blood, Lipidomics, Fish oil, Nutrition, Omega-3-index, Omega-3 long-chain polyunsaturated fatty acids, Phosphatidylcholines, Supplementation

## Abstract

Optimal dietary intake of omega-3 long-chain polyunsaturated fatty acids (n3-LCPUFAs) is critical to human health across the lifespan. However, omega-3 index (O3I) determination is not routinely assessed due to complicated procedures for n3-LCPUFA analysis from the phospholipid (PL) fraction of erythrocytes. Herein, a high-throughput method for lipidomics based on multisegment injection-nonaqueous capillary electrophoresis-mass spectrometry was applied to identify circulating PLs as surrogate biomarkers of O3I in two randomized placebo-controlled trials. An untargeted lipidomic data workflow using a subgroup analysis of serum extracts from sunflower oil versus high-dose fish oil (FO)-supplemented participants revealed that ingested n3-LCPUFAs were primarily distributed as their phosphatidylcholines (PCs) relative to other PL classes. In both high-dose FO (5.0 g/day) and EPA-only trials (3.0 g/day), PC (16:0_20:5) was the most responsive PL, whereas PC (16:0_22:6) was selective to DHA-only supplementation. We also demonstrated that the sum concentration of both these PCs in fasting serum or plasma samples was positively correlated to the O3I following FO (*r* = 0.708, *P* = 1.02 × 10^−11^, n = 69) and EPA- or DHA-only supplementation (*r* = 0.768, *P* = 1.01 × 10^−33^, n = 167). Overall, DHA was more effective in improving the O3I (ΔO3I = 4.90 ± 1.33%) compared to EPA (ΔO3I = 2.99 ± 1.19%) in young Canadian adults who had a poor nutritional status with an O3I (3.50 ± 0.68%) at baseline. Our method enables the rapid assessment of the O3I by directly measuring two circulating PC species in small volumes of blood, which may facilitate screening applications for population and precision health.

## Graphical abstract

**Figure:**
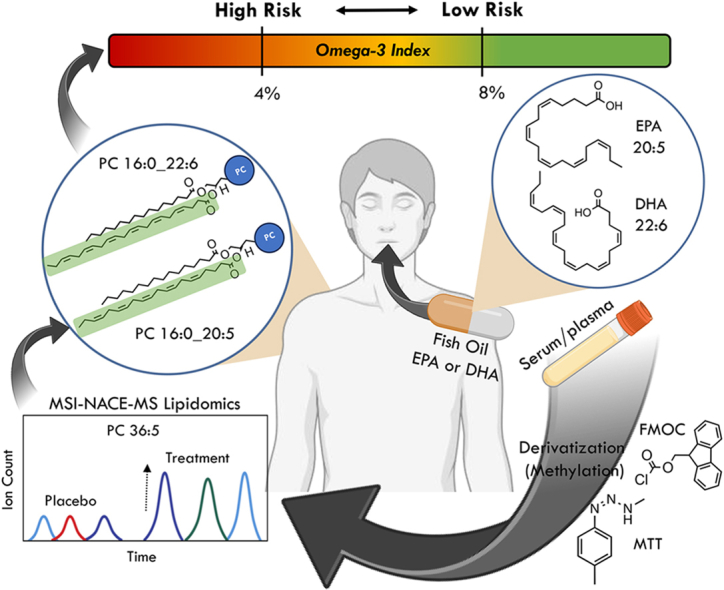

Evidence-based nutritional policies are urgently needed given an alarming increase in obesity and cardiometabolic disease burden worldwide (1). Public health guidelines have historically focused on lowering dietary fat intake (e.g., cholesterol, saturated fats) as a purported ‘heart healthy’ diet (2) rather than assessing overall diet quality (3). For instance, there is widespread deficiency of omega-3 long-chain polyunsaturated fatty acids (n3-LCPUFAs) as it comprises only a small fraction of total fats consumed in contemporary Western diets (4) since endogenous synthesis of this important class of FA is low (5). For these reasons, the American Heart Association Nutrition Committee recommends the consumption of oily fish/seafood as a marine source of dietary n3-LCPUFA up to twice a week to reduce cardiovascular disease risk (6). Unlike saturated or monounsaturated FAs, humans are unable to synthesize sufficient amounts of n3-LCPUFAs enriched within the cellular membrane of certain tissues/organs (e.g., retina, brain, heart), including DHA (22:6) and EPA (20:5) (7). Omega-3 FA nutrition impacts membrane composition and cellular function while also modulating inflammatory processes, such as the formation of resolvins and anti-inflammatory lipid mediators (8). Optimal intake of n3-LCPUFAs may also improve skeletal muscle function in older persons by enhancing amino acid-stimulated muscle protein synthesis rates and mitochondrial respiration kinetics (9).

Although dietary sources of n3-LCPUFAs are derived primarily from marine sources, the content of DHA and EPA in commonly consumed wild and farmed fish species varies widely (10). Alternatively, commercial fish oil (FO) dietary supplements offer a way to ensure adequate omega-3 FA nutrition together with emerging microalgae sources (11), and prescription EPA and/or DHA products (12). Yet, there have been conflicting results of n3-LCPUFAs in clinical trials in terms of their efficacy for cardiovascular disease protection (13). This outcome likely stems from inadequate dosage (<2 g/day) using impure formulations that do not target high-risk patients with hypertriglyceridemia and other comorbidities having a low baseline O3I status (14). For instance, high-dose prescription (4 g/day) icosapent ethyl treatment has been reported to reduce cardiovascular events in current and former smokers to levels similar to never smokers (15). However, prescription, supplemental and/or dietary intake of n3-LCPUFAs do not address excessive consumption of omega-6 FAs prevalent in processed foods (16), or differences in fatty acid desaturase activity (17) that contribute to variations in treatment response.

Nutritional epidemiological studies have relied on food frequency questionnaires to estimate omega-3 FA dietary fat intake for chronic disease risk assessment (18). Alternatively, biomarkers may offer a more reliable way to assess nutritional status (19) given between-subject differences in n3-LCPUFA bioavailability and metabolism, the variable content of omega-3 FAs in marine foods, and memory recall bias in self-reporting. In this case, the omega-3 index (O3I), defined as the erythrocyte EPA + DHA content from the phospholipid (PL) fraction as a mole percent to total fatty acids, represents a novel biomarker of coronary heart disease risk and sudden cardiac death independent of traditional risk factors (20). The O3I status can be stratified based on clinically defined cut-off values, where <4% is considered high risk, 4%–8% being intermediate risk, and low risk >8% for mortality from coronary heart disease (21), as confirmed in a meta-analysis from 10 cohort studies (22). For example, the mean O3I index for Canadian adults has been reported as 4.5% with less than 3% classified as having high cardioprotection (i.e., O3I >8%) that was dependent on age, ethnicity, fish consumption, supplement use, smoking status, and obesity (23). Although PL erythrocytes reflect habitual n3-LCPUFA intake patterns over a longer time interval (∼120 days) than other more dynamic PL class pools in circulation (24), a moderate to strong correlation of the O3I with EPA and DHA PL content measured in plasma or whole dried blood has been reported previously (21, 25). The disadvantages of the existing O3I index include the need to access erythrocytes, which are not widely available in biorepositories unlike other blood specimens (26, 27). Also, gas chromatography (GC) requires complicated sample handling procedures for O3I status determination after off-line PL fractionation by thin layer chromatography and their subsequent saponification into FA methyl esters, which is time consuming and less amenable to large-scale epidemiological studies (28). Also, there is a lack of standardization when reporting the O3I as varying number of FAs are measured by GC methods complicating comparative studies.

Herein, we performed an untargeted lipidomic analysis on fasted serum or plasma samples to identify circulating PL biomarkers of the O3I in young Canadian adults replicated in two placebo-controlled clinical trials using high-dose n3-LCPUFA (∼3 or 5 g/day) derived from FO, and purified EPA, or DHA only supplements (29, 30). We previously demonstrated that the sum of EPA and DHA measured as their nonesterified fatty acids (NEFAs) in serum ether extracts was associated with self-reported daily intake of fish/FO daily servings (31), where serum EPA was more responsive than DHA to high-dose FO ingestion (32). In this study, we aimed to identify intact PL species in circulation that may serve as surrogate biomarkers of the O3I when using multisegment injection-nonaqueous capillary electrophoresis-mass spectrometry (MSI-NACE-MS) (33). This multiplexed separation method offers a higher throughput platform for untargeted analysis of ionic lipids with stringent quality control when combined with a two-step chemical derivatization protocol (34). Our work introduces a more convenient and direct way to assess the O3I status using small volumes of blood without the need for off-line PL fractionation and saponification that is also applicable to other MS-based lipidomic instrumental platforms.

## Materials and methods

### Study designs, participants, and omega-3 FA supplementation trials

Both human n3-LCPUFA supplementation trials in this study obtained signed informed consent from all participants and abided by ethical principles of the Declaration of Helsinki. In the first discovery cohort, fasting serum samples were collected from participants in a randomized, double-blinded, placebo-controlled intervention study that investigated the effects of FO supplementation on attenuating skeletal muscle disuse atrophy following leg immobilization (29). This clinical trial was registered at the US National Library of Medicine (https://clinicaltrials.gov/) as NCT03059836 and approved by the Hamilton Integrated Research Ethics Board. Briefly, this study comprised a cohort of healthy young women with a mean age of 22 years (range: 19–31 years) and BMI of 24 kg/m^2^ (range: 18–26 kg/m^2^) recruited locally from the Hamilton area. All participants received either a high-dose FO (3.0 g EPA and 2.0 g DHA daily; n = 9) or a placebo control based on an isoenergetic and volume equivalent sunflower oil (SO) daily (n = 9). Repeat fasting serum samples were collected from participants at baseline and after 28, 42, and 56 days of the intervention. All serum samples were then stored frozen at −80°C. Further details on blood collection, participant selection and exclusion criteria, and erythrocyte PL omega-3 FA analysis for O3I determination are described elsewhere (29). Briefly, lipids were extracted from red blood cells using the Folch method (35) in chloroform-methanol (2:1 vol.) containing butylated hydroxytoluene (BHT, 0.01% vol.) as an antioxidant and heptadecanoic acid as an internal standard. Thin-layer chromatography silica plates isolated PL fractions (Silica Gel 60, 0.22 mm; Merck, Kenilworth, NJ) using heptane:isopropylether:acetic acid (60:40:3 vol.) as the elution solvent. Gel bands were scraped off the plate and transferred into screw cap tubes for transmethylation with BF_3_ in methanol. Fatty acid methyl esters (FAMEs) were then dissolved in hexane and analyzed using a Hewlett-Packard 5890 Series II GC with flame-ionization detection while using a Varian CP-SIL capillary column (100 m, internal diameter of 0.25 mm) (Palo Alto, CA). These measurements were then used to calculate the O3I by taking the sum of quantified EPA and DHA relative to the total of 17 saturated, monosaturated, and polyunsaturated FAs in fasting serum samples.

In the second validation cohort, fasting plasma samples were collected from participants in a randomized, double-blinded, multi-arm, placebo-controlled parallel group trial comparing the effects of supplementing using either ∼3 g/day EPA, DHA, or olive oil (OO) over a 90-day period (30). This study was approved by the Research Ethics Board at the University of Guelph. Participant characteristics (sex, age, BMI etc.) and blood draws for O3I assessment were obtained for all participants (n = 83) on study day visits. Purified EPA (KD-PUR EPA700TG) and DHA (KD-PUR DHA700TG) oils, as well as OO, were obtained from KD Pharma (Bexbach, Germany) with EPA and DHA in their triglyceride forms. The FA content of these supplements was previously reported to be 75.7% ± 0.01% for oleic acid (18:1) in the OO supplement, 74.7% ± 0.09% EPA and 0.55% ± 0.01% DHA in the EPA supplement, and 72.3% ± 1.3% DHA and 1.05 ± 0.11% EPA in the DHA supplement (36). All capsules contained 0.20% volume tocopherol to prevent oxidation of polyunsaturated lipids. Exclusion criteria included use of FO supplements within the previous 3 months, >2 servings of fish/seafood or other omega-3 FA-rich products per week, prescribed medication use (except oral contraceptives), current smoking, and history of cardiovascular disease. Participants were assigned *via* block randomization with stratification by sex to one of the three treatment arms, namely OO supplement (n = 27), EPA supplement (n = 28), and DHA supplement (n = 28). Participants were instructed to maintain regular exercise and dietary habits throughout the study. After overnight fasts, participants were subject to blood sampling at the Human Nutraceutical Research Unit at the University of Guelph before (baseline) and after (endpoint) of the 90-days intervention period. Blood was collected into EDTA-treated vacutainers, which was used to isolate plasma and erythrocytes. Samples were separated by centrifugation at 700 *g* at 4°C for 15 min. A similar protocol was performed for FAME analysis from erythrocyte PL extracts following fractionation and saponification using GC-FID with normalization to heptadecanoic acid as internal standard (30). In this case, the O3I was calculated by taking the sum of EPA and DHA relative to the sum of 15 saturated, monosaturated, and polyunsaturated FAs in fasting plasma samples.

### Sample workup, extraction, and derivatization procedure for MSI-NACE-MS

Fasting serum and plasma samples were subject to a two-step chemical derivatization protocol using 9-fluorenylmethyoxycarbonyl chloride (FMOC) and 3-methyl-1-*p*-tolyltriazene (FMOC/MTT) as described elsewhere (34). This reaction was introduced as a more convenient alternative to diazomethane to improve separation resolution and ionization efficiency by converting zwitter-ionic PL species that comigrate close to the electroosmotic flow (EOF) into methylated phosphatidylcholines (PCs) and SMs with a permanent positive charge. Briefly, in a glass sample vial, a 50 μl aliquot of serum/plasma sample was subject to a methyl-*tert*-butyl ether (MTBE) extraction, where 100 μl of methanol with 0.01% vol of BHT as antioxidant and PC 32:0[D62] as internal standard were first added, and samples were then mixed to induce protein precipitation. Next, 250 μl of MTBE was added and mixed prior to adding 100 μl of deionized water to induce phase separation. Samples were then centrifuged at 4,000 *g* at 4°C where then 200 μl of the organic layer was transferred into a new glass vial and dried down. Next, 100 μl of 0.85 mmol/L FMOC in chloroform containing PC 36:0[D70] as a second internal standard was added to dried serum/plasma extracts and mixed for 5 min at room temperature before drying down again. Next, 50 μl of MTBE containing 450 mmol/L of MTT was added to the glass vial with the lid sealed with Teflon tape. This vessel was then heated to 60°C for 60 min. Once the reaction was complete, the solution was dried down and then subject to a back extraction, where 100 μl of methanol was added, followed by 250 μl of hexane and then 200 μl of deionized water before centrifuging for 10 min at 4,000 *g* at 4°C. Then, 200 μl of the upper hexane layer was transferred out and dried down. Once completely dried, all samples were subsequently reconstituted in 50 μl containing acetonitrile/isopropanol/water (70:20:10 vol.) with 10 mmol/L ammonium formate and benzyltriethylammonium chloride as a third internal standard. All three internal standards had a final concentration of 5.0 μmol/L in the final plasma/serum extract, where PC 32:0[D62] was used for data normalization of methylated PCs to improve method precision based on their relative peak areas and relative migration times (RMT). Overall, derivatization yields of about 90% were achieved for quantitative analysis of methylated PCs by MSI-NACE-MS using a reference human plasma sample (34).

In order to expand overall lipidome coverage, lipid ether extracts were also analyzed directly without methylation, namely acidic/polar PL classes, including lysophosphatidylcholines (LPCs), phosphatidylethanolamines (PEs), lysophosphatidylethanolamines (LPEs), phosphatidylinositols (PIs), and NEFAs when using MSI-NACE-MS under negative ion mode as described elsewhere (33, 34). Briefly, a 50 μl aliquot was first subjected to MTBE extraction where 100 μl of MeOH containing 0.01% volume BHT was added to samples containing deuterated myristic acid, FA 14:0[D27] as an internal standard. Following rigorous shaking, phase separation induced by adding water, where samples were centrifuged to sediment protein at 4,000 *g* at 4°C for 30 min. The formation of a biphasic solution allowed for the top, lipid-rich ether layer to be extracted at a fixed volume (200 μl), where it was then dried under a gentle stream of nitrogen gas at room temperature. The dried extracts were then concentrated 2-fold after reconstitution in 25 μl acetonitrile-isopropanol-water (70:20:10 vol) containing 10 mM ammonium acetate and 50 μmol/L of deuterated stearic acid , FA 18:0[D35] as a second internal standard. However, FA 14:0[D27] was used for data normalization of acidic lipids to improve method precision.

### Untargeted and targeted lipidomics of serum/plasma ether extracts

An Agilent 6230 TOF mass spectrometer equipped with a coaxial sheath liquid ESI ionization source was used with an Agilent G7100A capillary electrophoresis (CE) unit for all experiments (Agilent Technologies Inc.). To supply a sheath liquid during electrophoretic separations, an Agilent 1260 Infinity isocratic pump delivered a solution containing 80% volume methanol with 0.1% volume formic acid at a flow rate of 10 μl/min into the sprayer. All separations were performed using bare fused-silica capillaries with an internal diameter of 50 μm, outer diameter of 360 μm, and total length of 110 cm (Polymicro Technologies Inc.). Electrophoretic separations were performed with an applied voltage of 30 kV with the capillary cartridge set at 25°C while using an isocratic pressure of 10 mbar (1 kPa). The background electrolyte (BGE) was 35 mmol/L ammonium formate in 70% volume acetonitrile, 15% volume methanol, and 5% volume isopropanol with an apparent pH of 2.3 that was adjusted by the addition of formic acid. Derivatized ether extracts were injected hydrodynamically at 50 mbar (5 kPa) alternating between 5 s for each sample plug and 40 s for the background electrolyte spacer plug to total seven discrete samples that were analyzed within 30 min for a single experimental run. Repeat QC samples were created by pooling samples from each study cohort, which were then introduced in a randomized position for each MSI-NACE-MS run to assess the technical precision of the method in both FO (n = 13) and EPA or DHA (n = 29) supplementation trials. All methylated lipid extracts were analyzed in positive ion mode acquisition with a V_cap_ at 3,500 V with full-scan data acquisition over the range of (*m/z* 50–1,700). Acidic lipids without derivatization were analyzed directly by MSI-NACE-MS under negative ion mode, which was performed only for the pooled subgroup analysis in the discovery FO trial. This instrumental configuration used a sheath liquid of 80% volume methanol with 0.5% volume ammonium hydroxide delivered at a flow rate of 10 μl/min using a CE-MS coaxial sheath liquid interface kit. The separations were performed on the same bare fused-silica capillaries with internal diameter of 50 μm, outer diameter of 360 μm, and total length of 95 cm. The applied voltage was set to 30 kV at 25°C for CE separations while applying an isocratic pressure of 20 mbar (2 kPa). The BGE consisted of 35 mM ammonium acetate in 70% volume acetonitrile, 15% volume methanol, and 5% volume isopropanol with an apparent pH of 9.5 that was adjusted using the addition of 12% vol ammonium hydroxide. These underivatized serum ether extracts from the FO discovery trial were injected hydrodynamically at 50 mbar (5 kPa) alternating between 5 s for each sample and 40 s for the BGE spacer for a total of seven discrete samples that were analyzed within a 30 min run (32, 33, 37). The TOF was operated in negative ion mode acquisition with V_cap_ at 3,500 V for full-scan data acquisition over the range of (*m/z* 50–1,700).

Overall, untargeted lipid profiling was performed on pooled serum extracts in a subgroup analysis of participants from the FO intervention trial when using MSI-NACE-MS under positive and negative ion modes. This was followed by a targeted lipidomic analysis and subsequent validation of lead candidate PC biomarkers responsive to n3-LCPUFA supplementation in both FO and EPA- or DHA-only placebo-controlled trials when using MSI-NACE-MS under positive ion mode following FMOC/MTT derivatization. Structural elucidation of putative PC biomarkers of the O3I were performed by collision-induced dissociation experiments when using a single injection format in CE coupled to a 6550 quadrupole-time of flight-mass spectrometer system (Agilent Technologies Inc.) at different collision energies under positive and negative ion mode as described elsewhere (32, 33, 37). Access to a purified reference standard for PC 16:0/22:6 (Toronto Research Chemicals, Toronto, ON) was available to confirm the likely molecular structure of PC 38:6 after spiking in pooled plasma (i.e., comigration) together with a comparison of the relative intensity of FA product ions using MS/MS under negative ion mode. However, lack of access to other lipid standards, including PC 16:0/20:5 and potential positional isomers (e.g., PC 22:6/16:0), prevented the reporting of definitive lipid molecular structures for these lipids in this study. Further details on the methodology used in this study are summarized in a reporting checklist from the Lipid Standards Initiative (https://doi.org/10.5281/zenodo.8339260).

### Data processing and statistical analysis

All MSI-NACE-MS data were analyzed using Agilent MassHunter Workstation Software (Qualitative Analysis Version 10.0, Agilent Technologies, 2012). All molecular features were extracted in profile mode within a 10 ppm mass window where derivatized lipids were annotated based on their characteristic *m/z* corresponding to their molecular ion and RMT. The manually integrated peak areas obtained from the extracted ion electropherograms were normalized to PC 32:0[D62] (positive ion mode) or FA 14:0[D27] (negative ion mode) to determine relative peak areas and RMT for serum/plasma lipids. Extracted ion electropherograms were integrated after smoothing using a quadratic/cubic Savitzky-Golay filter (7 points). Absolute concentrations reported for selected PLs were estimated based on a serial dilution of NIST SRM-1950 human plasma when using MSI-NACE-MS (34) based on consensus concentrations reported in a multicenter lipidomics harmonization study (38). However, PC 38:6 was quantified directly using an external calibration curve normalized to PC 32:0[D62], whereas the response factor for PC 36:5 was estimated using a higher abundance surrogate lipid, PC 36:4 needed to attain adequate linear dynamic range after serial dilution of NIST-SRM 1950 human plasma (34). Least-squares linear regression and correlation plots were performed using Excel (Microsoft Office). Visualization of data, heat maps, and unsupervised principal component analysis were performed using MetaboAnalyst version 5.0 (39). Normality tests and nonparametric statistical analysis was performed using IBM SPSS version 23 (IBM), whereas MedCalc version 12.5.0 (MedCalc Software) was used to generate boxplots and control charts with exception of trajectory box plots (R Foundation for Statistical Computing). A two-way between and within mixed-model ANOVA (treatment × time) was used for assessing the impact of high-dose FO supplementation at three times points as compared to baseline. For the study involving DHA or EPA supplementation relative to OO as placebo, a Wilcoxon signed ranked test was performed to evaluate treatment effects after confirming non-normally distributed data. A Pearson correlation analysis was used to evaluate the association between lead candidate PCs in serum or plasma extracts as compared to the O3I based on erythrocyte membrane PLs.

## Results

### Sub-group analysis for identifying serum PLs responsive to FO intake

**Fig. 1:**
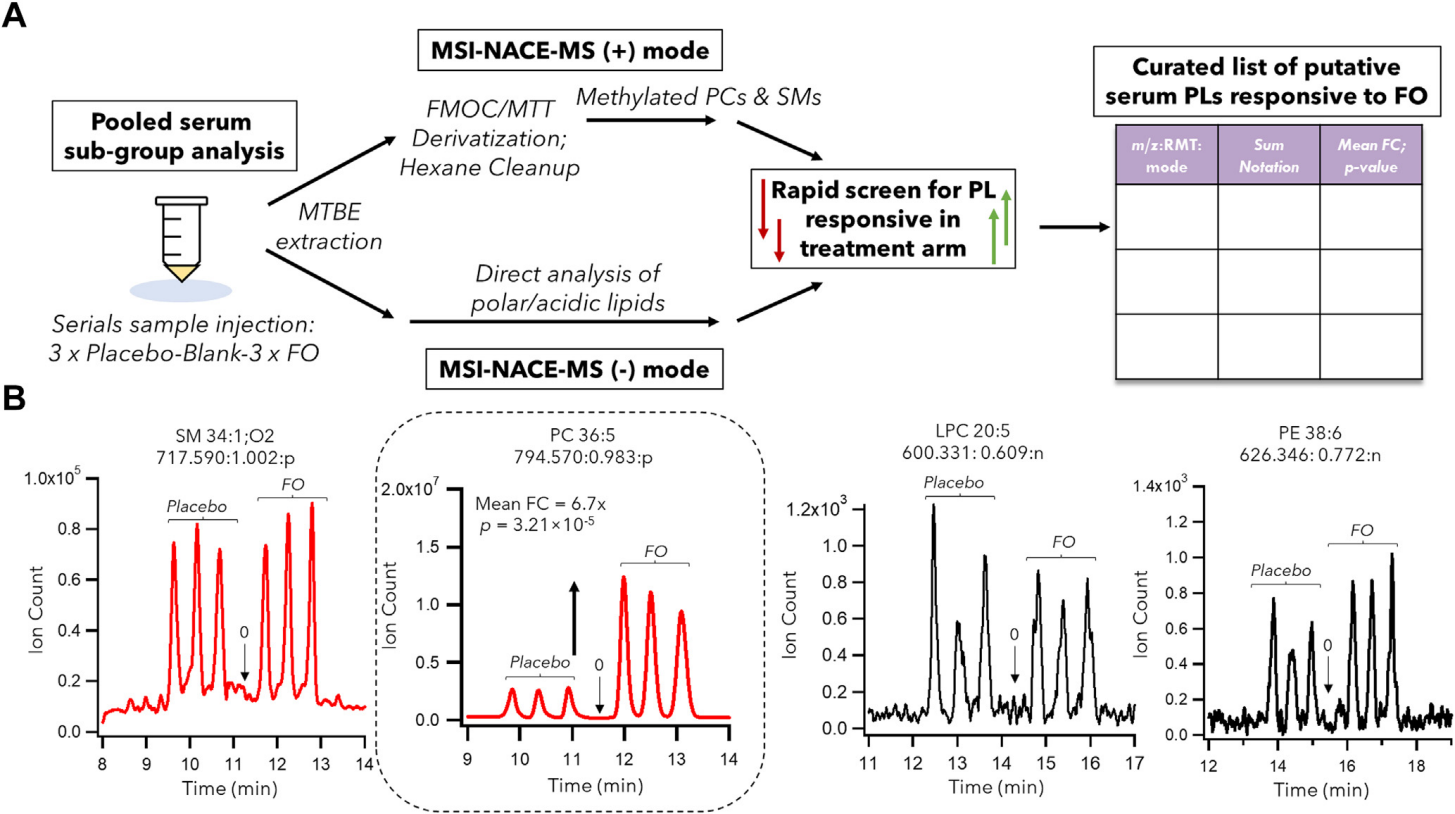
A: Overview of an accelerated data workflow for rapid identification of putative serum biomarkers of n3-LCPUFA intake following high-dose FO supplementation as compared to placebo/baseline when using MSI-NACE-MS with temporal pattern signal pattern recognition under two complementary configurations. B: Representative extracted ion electropherograms highlighting various PL classes/species that do not change following FO supplementation (e.g., SM 34:1;O2, LPC 20:5, PE 38:6) in contrast to specific serum PC species that undergo a notable increase after FO ingestion, such as PC 36:5. In all cases, a subgroup analysis was performed by MSI-NACE-MS under positive and negative ion modes when analyzing a serial injection of pooled serum samples from participants in the placebo/baseline and FO treatment arms in triplicate together with a blank extract as control. All lipids in serum extracts were annotated based on their accurate mass (*m/z*), relative migration time (RMT), and ionization mode (p or n). FO, fish oil; MSI-NACE-MS, multisegment injection-nonaqueous capillary electrophoresis-mass spectrometry; n3-LCPUFA, omega-3 long-chain polyunsaturated fatty acid; PC, phosphatidylcholine; PL, phospholipid.

We initially performed an untargeted screen for serum PLs associated with n3-LCPUFA supplementation based on an analysis of pooled serum extracts from all participants in the placebo/baseline as compared to the FO treatment arm (EPA, 3 g/day + DHA, 2 g/day). These two subgroups of samples were analyzed in triplicate with a blank extract to rapidly identify differentiating PL species following high-dose FO ingestion using two complementary MSI-NACE-MS configurations as shown in Fig. 1A. This strategy takes advantage of a serial injection of seven serum extracts within a single analytical run by MSI-NACE-MS in positive or negative ion mode when using temporal signal pattern recognition (40, 41) and enables reliable credentialing of lipid features responsive to FO ingestion after rejecting spurious signals, background ions, and a majority of nonresponsive PLs. Overall, serum PCs and SMs as their cationic methylphosphate esters were preferentially analyzed by MSI-NACE-MS under positive ion mode after FMOC/MTT derivatization, whereas electrically neutral lipid classes (e.g., triacylglycerides, cholesterol esters) comigrate with the EOF (34). This two-step chemical derivatization procedure relies on MTT as a less hazardous methylating reagent to diazomethane avoiding the need for blast shields and personal protective equipment (42). However, FMOC was first added prior to MTT to protect certain PLs having reactive primary amine head groups (e.g., PEs) thereby avoiding the generation of permethylated isobaric interferences to analogous PCs (34). Otherwise, direct analysis of PEs and other acidic/polar lipid classes (e.g., PSs, PAs, LPCs) that did not benefit from methylation or had a poor recovery in hexane was performed by MSI-NACE-MS under negative ion mode conditions to expand overall lipidome coverage (33, 34).

For example, Fig. 1B illustrates four representative PL classes from pooled subgroups of serum extracts annotated by their accurate mass, relative migration time, ionization mode (*m/z*:RMT:p or n), and their sum composition, including SM 34:1;O2, PC 36:5, LPC 20:5, and PE 38:6. Importantly, these serum-derived lipids were not prone to sample carry-over effects when using serial sample injections in MSI-NACE-MS as demonstrated by a blank extract analyzed within the same run. As expected, serum PLs with only a single degree of unsaturation, such as SM 34:1;O2, did not exhibit a change in response following FO supplementation as compared to baseline/placebo. Two other PL classes measured directly from serum extracts by MSI-NACE-MS under negative ion mode, including a putative EPA-containing LPC 20:5 and a DHA-containing PE (PE 38:6), also did not change (*P* > 0.05) following FO intake. In contrast, PC 36:5 exhibited a striking 10-fold change (FC) average increase from baseline in response to FO supplementation. Similarly, underivatized PC 38:6 and PC 36:5 measured directly under negative ion mode were independently demonstrated to undergo a similar response increase following FO supplementation (supplemental Fig. S1). As zwitter-ionic PCs migrate close to the EOF under these conditions, resolution was poor, and their ion responses were prone to matrix-induced ion suppression that lowered overall sensitivity. For these reasons, we applied a quantitative methylation reaction as a charge-switching derivatization strategy in lipidomics to improve their separation resolution and detectability when using MSI-NACE-MS under positive ion mode (34). This approach also reduces isobaric interferences among distinct lipid classes based on differences in their apparent electrophoretic mobility, such as methylated SMs and PCs (34). Other DHA-containing PCs, such as PC 38:6, exhibited a more modest increase after FO intake, as well as EPA and DHA as their NEFAs (31, 32). Overall, only six PC species likely containing DHA and EPA fatty acyls chains from a total of 84 annotated ionic lipids (supplemental Table S1) were classified as putative lipid biomarkers that increased following FO ingestion together with their NEFAs (*P* < 0.05) under fasting conditions. However, other ionic PL classes containing likely EPA or DHA (e.g., LPCs, PIs, PEs, LPEs etc.) were not found to be responsive to FO supplementation in our study. Also, certain serum PLs may comprise unresolved mixtures of isomers or isobars that lack specificity (e.g., PC 38:5), whereas other NEFAs derived from less abundant n3-LCPUFA in FO did not respond to supplementation, such as docosapentaenoic acid (22:5). Type-2 isotopic effects were also not a significant problem to correct for as most comigrating lipid isotopomers, notably for PC 35:5 and PC 38:6, lacked homologous PCs having an additional double bond (supplemental Fig. S2). As a result, we primarily focused on data integration of methylated serum PCs analyzed by MSI-NACE-MS under positive ion mode detection, notably top-ranked candidate biomarkers of omega-3 FA nutrition identified by this untargeted lipidomics screen involving pooled subgroups of participants prior to and following high-dose FO supplementation.

### Validation of serum PC panels associated with high-dose FO supplementation

**Fig. 2:**
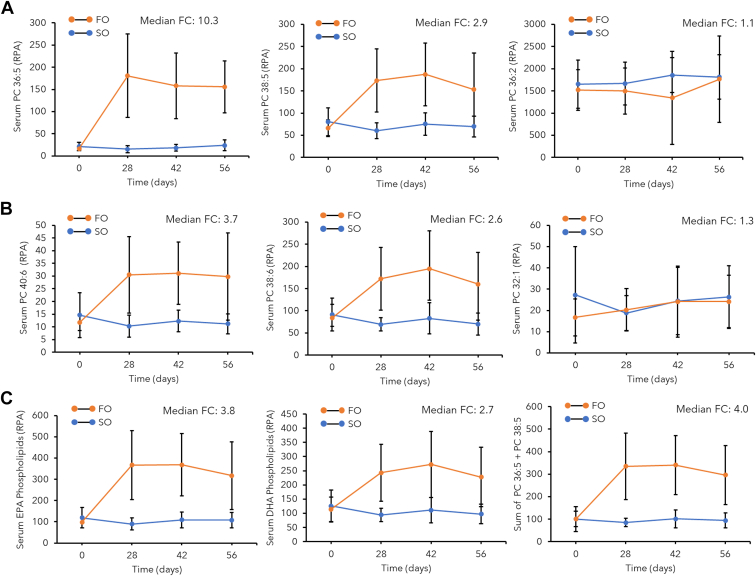
Serum PC trajectory plots measured in young women (n = 9) from baseline after ingesting high-dose fish oil (FO) supplement (3 g/day EPA+2 g/day DHA) as compared to sunflower oil (SO) as placebo over 56-day period. A: Highlights two top-ranked serum PCs (PC 36:5; PC 38:5) that were most responsive to FO intake unlike a linoleic acid containing PC as control (PC 36:2). B: Highlights two top-ranked serum PCs (PC 40:6; PC 38:6) that were most responsive to FO intake unlike an oleic acid containing PC as control (PC 32:1). C: A comparison of treatment response trajectories to high-dose FO intake based on the sum of three EPA (PC 36:5+PC 38:5+PC 40:5) or DHA (PC 36:6+PC38:6+PC 40:6) associated PCs as compared to the sum of only two EPA and DHA-specific lipid biomarkers (PC 36:5+PC 38:6). Error bars on plots represent ± 1s as the biological variance that increased after FO supplementation relative to baseline or placebo. Table 1 summarizes statistical outcomes when using a two-way repeat measures ANOVA mixed model from this data, whereas lipid responses were reported in terms of their relative peak area (RPA) with signals normalized to PC 32:0[D62] as an internal standard. EPA, eicosapentaenoic acid; PC, phosphatidylcholine.

**Fig. 3:**
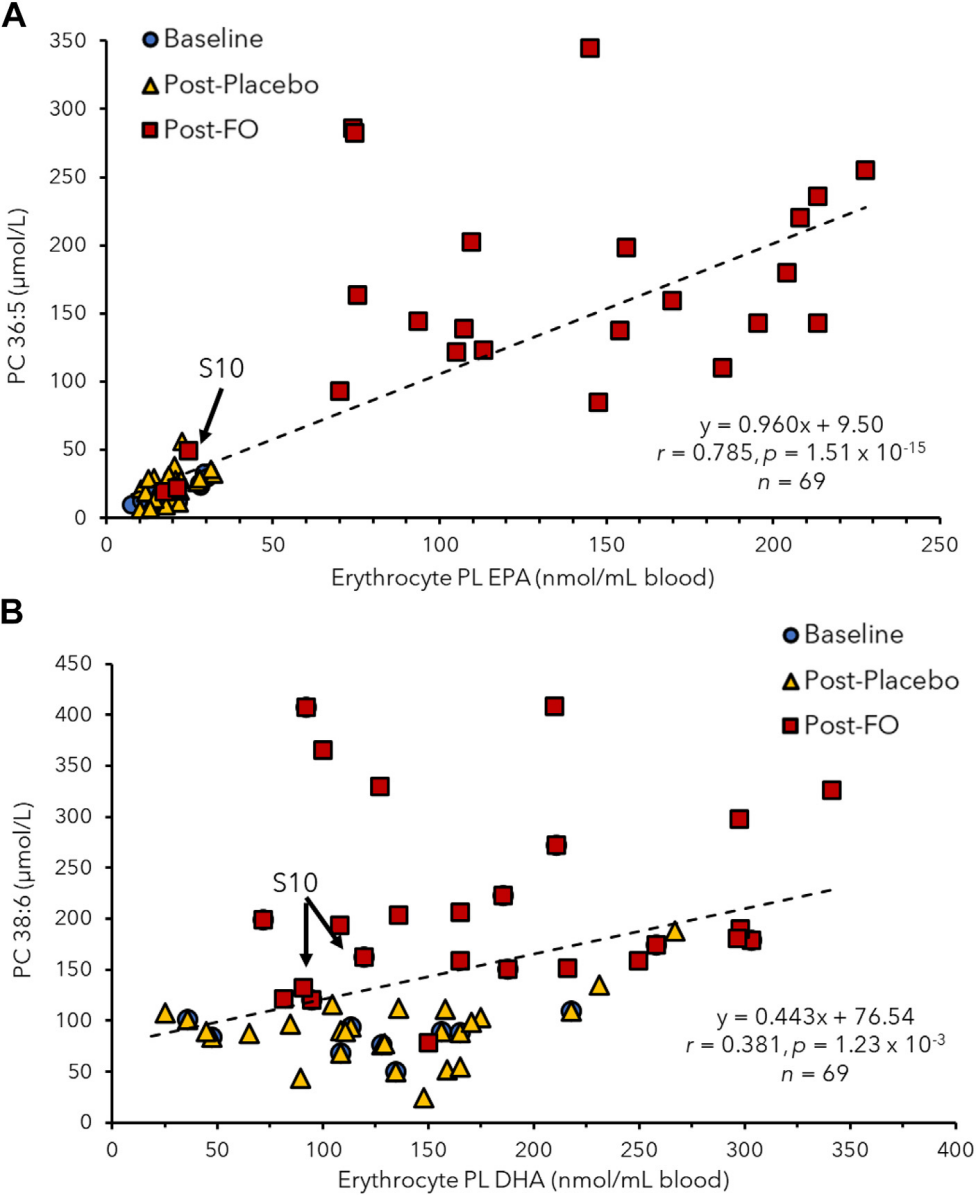
A scatter plot showing a strong linear correlation (*r* = 0.738, n = 69) between (A) a serum PC containing EPA (PC 36:5 or PC 16:0_20:5) with erythrocyte PL-derived EPA measurements that is more sensitive to treatment responses following FO supplementation at three time intervals (28, 42, and 56 days), including detection of dietary nonadherence for a participant (S10). B: There was a weaker linear correlation (*r* = 0.381, n = 69) for a serum PC containing DHA (PC 38:6 or PC 16:0_22:6) when compared to erythrocyte PL-derived DHA measurements. EPA, eicosapentaenoic acid; FO, fish oil; PC, phosphatidylcholine; PL, phospholipid.

Overall, 44 PC species were quantified consistently from all serum ether extracts in a cohort of young women (n = 8) at baseline and then at three time points following high-dose FO or SO placebo intervention over 56 days (supplemental Fig. S3A). A 2D principal component analysis scores plot with hierarchical cluster analysis heat map comprising 44 serum PCs following *glog*-transformation and autoscaling illustrates the overall data structure (supplemental Fig. S3B). Good technical precision was achieved from a repeat analysis of a pooled QC sample (median CV = 13%, n = 13) as compared to the biological (between-subject) variance in the serum lipidome for all participants (median CV = 49%, n = 69). Figure 2 depict time series trajectories for a series of top-ranked serum biomarkers associated with high-dose FO ingestion as compared to the intake of SO as placebo when using a repeat measures mixed 2-way ANOVA model (Table 1). As expected, elevated and steady-state levels of circulating EPA and DHA containing PCs as a single species or their sum were reached within 28 days in the FO treatment arm as compared to placebo. Since SO contains linoleic (FA 18:2) and oleic acid (FA 18:1) as major FA constituents, serum levels of PC 32:1 and PC 36:2 were also included as controls, but they showed no change (*P* > 0.05) in either placebo and FO treatments. Table 1 highlights that two circulating PC lipid species, namely the sum of PC 36:5 and PC 38:6, generated the greatest effect size (0.851) and statistical significance (*P* = 9.93 × 10^–7^) in response to high-dose FO supplementation relative to placebo unlike other larger PC panels (up to six) or single PC lipid species. Importantly, the sum concentration (μmol/L) of serum PC 36:5 and PC 38:6 exhibited a positive correlation (*r* = 0.714, *P* = 5.53 × 10^–12^) to the O3I derived from the wt% of EPA and DHA of PLs in erythrocyte membranes (supplemental Fig. S4). In fact, an improved correlation with greater sensitivity to FO intake was achieved for serum PC 36:5 + PC 38:6 as compared to EPA + DHA previously measured as their NEFAs (32). We tentatively identified these two circulating lipid biomarkers of the O3I as PC (16:0_20:5) and PC (16:0_22:6) following annotation of their MS/MS spectra in positive and negative ion mode detection (supplemental Fig. S5). Serum PC concentrations were also measured with external calibration curves (supplemental Fig. S6) using a purified lipid standard (PC 16:0/22:6) or estimated using a surrogate PC (PC 36:4 for PC 36:5) following serial dilution of NIST-SRM 1950 human plasma based on consensus concentrations reported in a lipidomics harmonization study (34). Temporal changes in lipidome profiles also demonstrated that serum PCs containing EPA fatty acyl chains responded to FO supplementation more than DHA containing PCs. This was reflected by a stronger association for serum PC 36:5 concentrations (μmol/L) and EPA erythrocyte PL content (nmol/ml) (*r* = 0.785, *P* = 1.51 × 10^–15^) than serum PC 38:6 and DHA erythrocyte PL content (*r* = 0.381, *P* = 1.23 × 10^–3^) as shown in Fig. 3. The greater sensitivity of serum PC 36:5 following FO intake was also useful to screen for likely dietary nonadherence of a participant (32), who was excluded from subsequent statistical analyses in this study.

**Table 1:** Summary of responsive serum PCs associated with high-dose FO intake as classified by a repeat measures two-way mixed-model ANOVA as compared to placebo (SO)

Serum PCs	Within-Subject Effects				Between-Subject Effects			
	*F*	*p* value^a^	Effect Size^b^	Study Power	*F*	*P* value	Effect Size^b^	Study Power
PC 36:5 + PC 38:6^c^	6.89	7.81 × 10^−4^	0.346	0.965	74.1	9.93 × 10^−7^	0.851	1.000
Total EPA PCs^d^	6.76	8.83 × 10^−4^	0.342	0.962	54.2	6.00 × 10^−6^	0.806	1.000
PC 36:5	6.69	9.42 × 10^−4^	0.340	0.960	33.4	6.30 × 10^−5^	0.720	1.000
Total omega-3 PCs^e^	6.30	1.37 × 10^−3^	0.326	0.949	14.8	2.00 × 10^−3^	0.533	0.945
Total DHA PCs^f^	5.39	3.36 × 10^−3^	0.293	0.909	32.8	7.00 × 10^−5^	0.716	1.000
PC 38:5	5.12	4.43 × 10^−3^	0.282	0.893	39.0	3.00 × 10^−5^	0.750	1.000
PC 38:6	4.94	5.27 × 10^−3^	0.276	0.882	20.2	6.01 × 10^−4^	0.609	1.000
PC 40:6	4.51	8.26 × 10^−3^	0.258	0.848	25.6	2.17 × 10^−4^	0.664	0.997
PC 36:1	1.37	2.66 × 10^−3^	0.095	0.336	0.01	9.35 × 10^−1^	0.001	0.051
PC 36:2	0.23	8.72 × 10^−1^	0.018	0.090	0.16	6.95 × 10^−1^	0.012	0.066

a Mixed-model ANOVA applied where data sphericity satisfied using Mauchly’s test of sphericity, including an FO treatment and placebo treatment at three time points (28, 42, and 56 days) from baseline following supplementation.

b Effect size based on partial eta squared.

c Serum lipids associated with PC 36:5 and PC 38:6 are PC (16:0_20:5) and PC (16:0_22:6), respectively.

d Three putative EPA-containing PCs comprise the sum of PC 36:5 + PC 38:5 + PC 40:5. Serum PC 38:5 subsequently determined to constitute an unresolved mixture of two isobaric PCs (16:0_22:5 and 18:1_20:4).

e Six putative omega-3-containing PCs comprise the sum of PC 36:5 + PC 38:5 + PC 40:5 + PC 36:6 + PC 38:6 + PC 40:6.

f Three DHA-containing PCs comprise the sum of PC 36:6 + PC 38:6 + PC 40:6.

### Validation of serum PC biomarkers of O3I status following DHA or EPA intake

As the high-dose FO trial relied on an unequal mixture of n3-LCPUFAs in a modest number of women, we next aimed to further validate lead candidate PC biomarkers of O3I status in an independent trial involving a larger cohort (n = 83) using purified EPA- or DHA-only supplements at the same dosage level (∼3 g/day over a 56-day period) relative to OO as the placebo. In addition, we sought to confirm whether the same lipids can be related to the O3I in a different blood specimen type, namely human plasma (EDTA as anticoagulant) rather than serum. This cohort comprised young, normal weight, nonsmoking Canadian adults of both sexes who had a different (*P* = 5.67 × 10^–3^) baseline O3I status of (3.77 ± 0.63%) and (3.34 ± 0.76%) for women (n = 43) and men (n = 40), respectively (supplemental Table S2). Also, the mean O3I status at baseline for all participants was (3.50 ± 0.68% ranging from 1.87% to 5.21%) with 75% of participants having an O3I <4%. High-dose EPA and DHA intake significantly increased their average O3I status from baseline to (8.30 ± 1.21%) and (6.49 ± 1.17%), respectively, as compared to OO placebo (3.61 ± 0.60%) after 90 days. As a result, DHA more effectively increased the O3I than EPA supplementation as reflected by 71% versus 11% of participants achieving a low cardiovascular risk profile of O3I >8%, respectively.

**Fig. 4:**
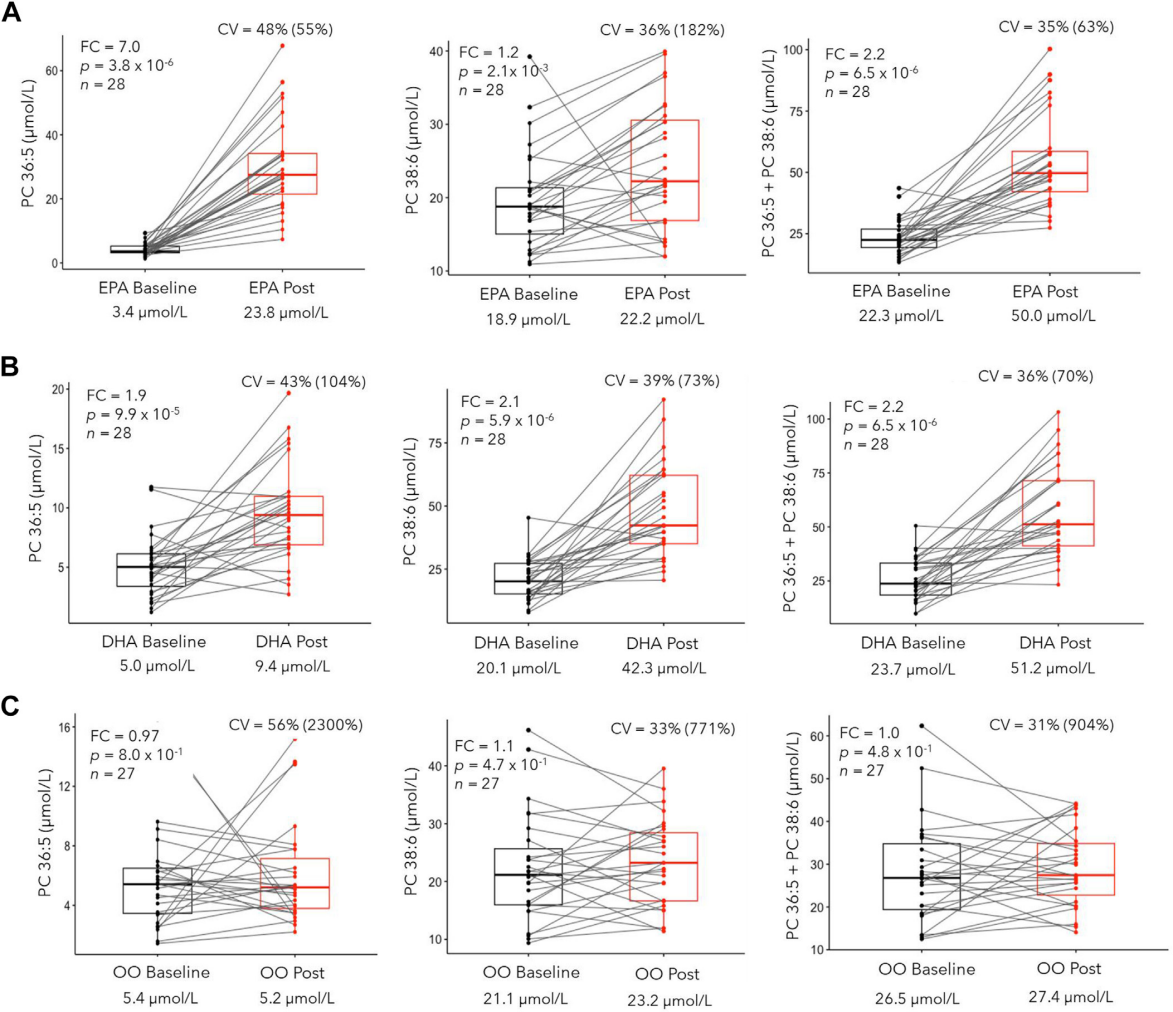
Representative spaghetti plots depicting responses of top-ranking plasma PLs after treatment with either (A) EPA, (B) DHA, or (C) olive oil (OO) as placebo based on single lipid biomarkers and their sum. Overall, PC 36:5 or PC 16:0_20:5 serves as a circulating biomarker most sensitive to EPA only supplementation as compared to PC 38:6 or PC 16:0_22:6 following DHA only ingestion at a similar dosage (3 g/day) after a 90-day period. No differences were measured in EPA or DHA-containing plasma PCs after ingestion of OO. Median concentrations for plasma PCs at baseline and following treatment are reported along with between-subject variation (as a mean CV) in responses following treatment (or their difference from baseline in brackets). EPA, eicosapentaenoic acid; PC, phosphatidylcholine; PL, phospholipid.

After identifying several n3-LCPUFA containing PC species and panels responsive to FO supplementation in the subgroup screen (supplemental Table S1) and full analysis (Table 1), we subsequently performed a targeted lipidomic analyses of these PC biomarker candidates in a second independent placebo-controlled EPA- and DHA-only trial. As expected, the same circulating PCs responded to this specific n3-LCPUFA dietary intervention, notably PC 36:5 from a median baseline of 3.4 μmol/L to 23.8 μmol/L (median FC ∼7.0) following EPA only ingestion as shown in Fig. 4A. The spaghetti box plot also highlights considerable treatment response variability between-subjects, which had a mean CV = 48% for PC 36:5 concentrations measured after EPA supplementation alone or a mean CV = 55% based on plasma concentration changes from baseline for individual participants. In contrast, there was a modest increase (∼17%) in DHA-containing PC 38:6 concentrations from baseline after EPA intake from 18.9 μmol/L to 22.0 μmol/L with much larger between-subject treatment response variations (mean CV = 182%). This also coincided in a lower treatment response (median FC ∼2.2) overall when measuring the sum of plasma PC 36:5 and PC 38:6 concentrations following high-dose EPA supplementation.

In contrast, ingestion of a high-dose DHA-purified supplement elicited a more attenuated increase in plasma PC 38:6 (median FC ∼2.1) from 20.1 μmol/L to 42.3 μmol/L that was similar in magnitude to the EPA-containing PC 36:5 (∼88% increase from baseline) as highlighted in Fig. 4B. This was likely due to the higher (∼4.7-fold) baseline concentrations for plasma PC 38:6 as compared to PC 36:5, thereby being less sensitive to high-dose DHA supplementation. As a result, the sum concentration for plasma PC 36:5 and PC 38:6 generated a similar overall treatment response following DHA intake. As the constituents of OO consisted primarily of linoleic acid and oleic acid, Fig. 4C confirmed no change in either PC 36:5, PC 38:6, or their sum in the placebo arm (*P* > 0.05). However, a modest increase (∼1.2-fold, *p* ∼ 0.004) in plasma PC 36:1 and PC 38:2 was measured from baseline following OO intake (supplemental Fig. S7). Yet, this effect was much lower in magnitude than treatment responses involving the two omega-3 FA containing PCs following high-dose EPA or DHA intake.

### Two circulating PCs as surrogate biomarkers of the O3I

**Fig. 5:**
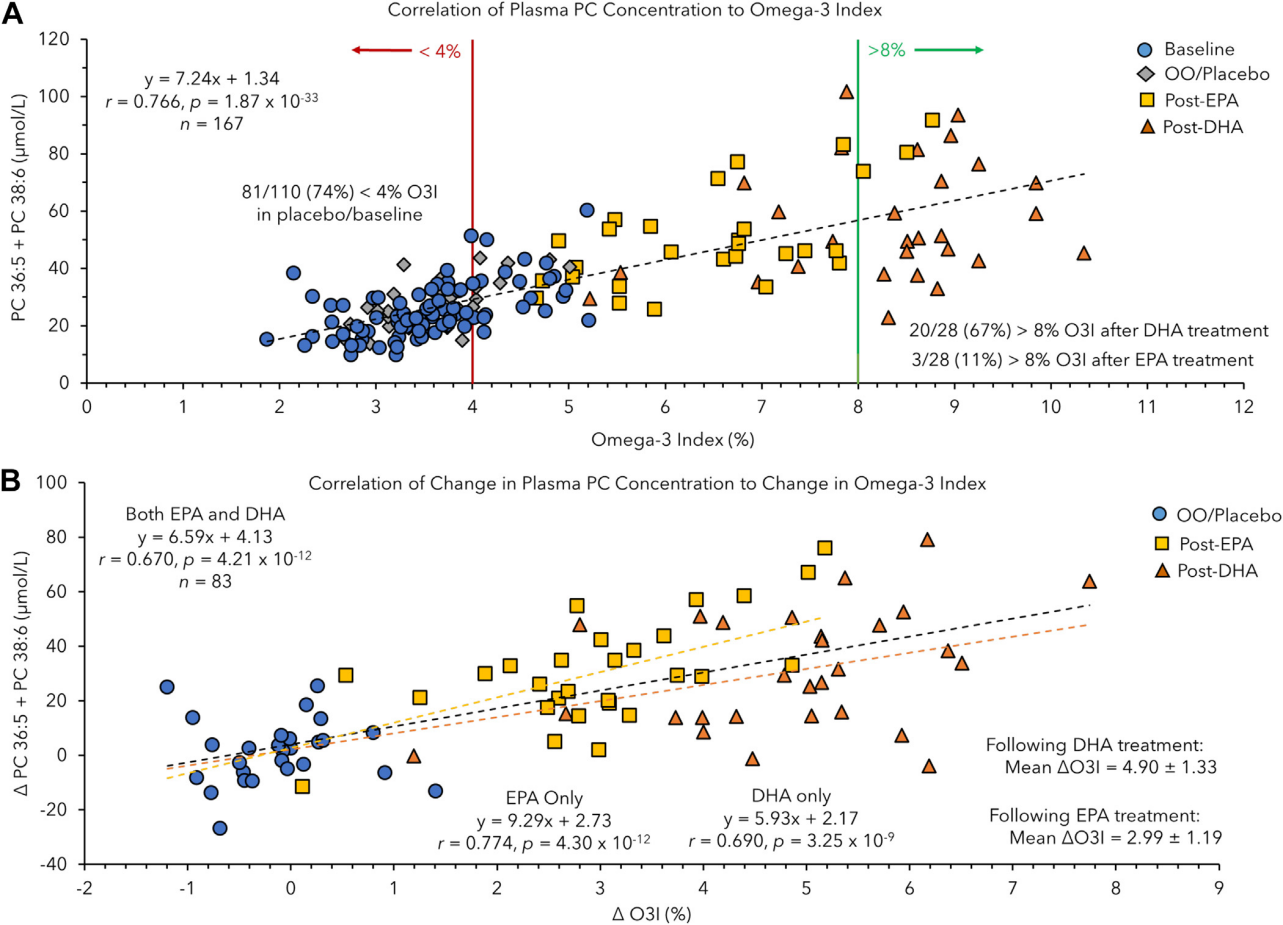
A: Scatter plot showing strong correlation (*r* = 0.764, *P* = 3.0 × 10^−33^) of the O3I to plasma concentrations of the sum of PC 36:5 and PC 38:6 at baseline/placebo and following EPA or DHA-only supplementation (n = 167). Established O3I cut-off intervals are stratified into three categories, where <4%, 4%–8%, and >8% are defined as high-, intermediate- and low-risk profiles for cardiovascular events. B: Overall, high-dose DHA supplementation contributed to a greater treatment response than EPA intake as depicted in the correlation plot between the sum concentration for these two plasma PCs to the change in O3I status from baseline for individual participants. EPA, eicosapentaenoic acid; O3I, omega-3 index; PC, phosphatidylcholine.

We next aimed to explore whether circulating PCs may serve as potential surrogate measures of erythrocyte PL-derived O3I while also reflecting intake of high-dose FO intake or purified supplements of either EPA or DHA. While the total sum of all six n3-LCPUFA containing PC species demonstrated a moderate correlation (*r* = 0.636) to the O3I, statistical outcomes were improved when using fewer PCs within the panel (supplemental Table S3). Similar to the outcomes reported from the high-dose FO trial, the strongest correlation to the O3I in this cohort was achieved using the sum concentration for plasma PC 36:5 and PC 38:6, representing two of the most abundant circulating EPA and DHA containing PL species in human blood (34). Figure 5A depicts a correlation plot for plasma PC 36:5 + PC 38:6 based on their absolute concentrations (μmol/L) as a function of O3I (*r* = 0.768, *P* = 1.01 × 10^−33^), which highlights a distinct enhancement in omega-3 FA nutrition after 90 days of supplementation. Importantly, most participants (∼74% or 81/110 with a mean O3I of 3.56%) had a high-risk O3I profile (<4%) at baseline and after OO supplementation, whereas only 26% were classified as having a moderate risk category (4%–8%) with not a single participant having an O3I >8%. In contrast, 67% and 11% of participants following intake of 3.0 g of EPA or DHA had their O3I status changed into a low-risk profile for cardiovascular health (>8% O3I), respectively. Although EPA was less efficacious in increasing the O3I than DHA at the same dosage level, all participants improved to at least a moderate risk category (4%–8%). Also, reporting the fraction (%) of PC 36:5 + PC 38:6 normalized to a total of 44 plasma PCs measured by MSI-NACE-MS, provided only a modest improvement in its association with the O3I (*r* = 0.788, *P* = 1.25× 10^−36^) as compared to the absolute concentration for two PCs alone (supplemental Fig. S8). Additionally, we explored these differential treatment response outcomes by considering EPA- and DHA-specific correlations to plasma PC 36:5 + PC 38:6 concentration as a function of differences in the O3I status from baseline as depicted in Fig. 5B. As expected, DHA supplementation alone contributed to a 63% greater relative efficacy overall (ΔO3I = 4.90 ± 1.33%) as compared to participants ingesting a similar dose of EPA (ΔO3I = 2.99 ± 1.19%). This difference in treatment response was also captured by comparing the slopes determined from the correlation of DHA- (slope = 5.93) and EPA- (slope = 9.29) only supplement subgroups using a least-squares linear regression model. This approach may enable correction for the attenuated DHA treatment response relative to EPA within the circulating PC lipid pool as required to estimate their composition within erythrocyte membranes that itself serves as a proxy for cardiac tissue (20, 21). Overall, there was a modest sex-dependence (*p* ∼0.02) found in measured changes in plasma PC 36:5 + PC 38:6 concentrations and the O3I from baseline (supplemental Table S4). Overall, this effect was more pronounced in females ingesting EPA who had greater increases in their circulating concentrations of n3-LCPUFA-containing PCs. In contrast, males who ingested DHA had greater changes in the O3I than females reflecting their lower baseline status.

## Discussion

Epidemiological studies of Greenland Inuit consuming a traditional diet rich in marine organisms first implicated greater n3-LCPUFA intake with a lower incidence of cardiovascular disease than Western dietary patterns (43). However, changes in diet and cultural practices have lowered the omega-3 FA nutritional status of contemporary Inuit coinciding with an epidemiological transition of greater chronic disease burden and psychological distress (44, 45). Several prospective studies in other populations have reported that low fish/seafood consumption and poor n3-LCPUFA nutrition is associated with higher all-cause and cardiovascular mortality (46, 47, 48, 49), with EPA demonstrating the strongest association independent of other risk factors (50). Indeed, clinical trials involving purified high-dose (∼3–4 g/day) EPA and its analogs provide growing evidence of its utility as an adjunct therapy for the prevention of major coronary events in high-risk patients (51, 52) by reducing circulating triglyceride levels as well as vascular inflammation as compared to DHA alone or DHA+EPA mixtures (53). Thus, EPA and DHA have overlapping and divergent effects on gene expression (54), membrane structure (55), lipogenesis (30), and cellular metabolism in subjects with chronic inflammation (56). As a result, objective biomarkers of n3-LCPUFA intake are urgently needed to measure these essential FAs during the lifespan as they are not reliably quantified by questionnaires given the variability in their amount, quality, and composition in dietary fats (57).

To date, a major challenge in using the O3I as a risk assessment tool in clinical medicine is the variety of analytical methods (e.g., specimen type, extraction procedure, fractionation etc.) used for measuring n3-LCPUFAs from different circulating lipid pools, including erythrocytes, plasma total lipids, plasma PL fraction, and whole blood (58, 59). Although the gold standard for O3I determination remains GC analysis of FAMEs from the PL fraction of erythrocytes isolated after thin-layer chromatography, this procedure is both time consuming and less amenable to high throughput screening (20, 21, 22). Also, the total number of reported fatty acids (up to 50) can vary widely between methods, which complicate standardization and data comparisons when reporting the sum of EPA and DHA as their wt% (47). Alternatively, ^1^H-NMR may enable the reliable estimation of O3I status in large-scale prospective studies based on the analysis of DHA% and non-DHA% plasma lipoproteins with a good mutual agreement to GC results (27). However, neither GC or NMR methods directly resolve and quantify specific intact lipid species in small volumes of blood specimens that are best achieved when using chromatographic, ion mobility or electrophoretic separations coupled to high resolution MS (60). Herein, we applied a high throughput lipidomic platform based on MSI-NACE-MS under two configurations that takes advantage of serial injection of seven serum/plasma extracts in a single analytical run (33, 34). MSI-NACE-MS allows for unique data workflows by encoding mass spectral information temporally within a separation when performing untargeted lipidomics. For instance, this approach was used to reliably authenticate and identify lipid features that increased following high-dose FO intake in a pooled subgroup analysis, which was subsequently validated in two randomized placebo-controlled trials, including EPA- or DHA-only supplementation. Although a two-stage FMOC/MTT derivatization procedure is required to generate cationic methylated PCs from serum/plasma ether extracts prior to MSI-NACE-MS analyses, this is far less hazardous than using diazomethane previously reported to improve the chromatographic performance as well as enhance the selectivity and sensitivity for glycerophospholipid and sphingolipid analyses by LC-MS/MS (42, 61). In general, MSI-NACE-MS offers better selectivity than HILIC-MS methods since polar/ionic lipids are resolved based on differences not only in their polar head group but also bond linkage and total acyl chain length that impact their apparent electrophoretic mobility (34). However, type-II isobaric interferences may occur if not verified or corrected for in complex biological samples due to comigration of PLs having differences in the number of double bonds (supplemental Fig. S2), which can be minimized with higher resolution mass analyzers and optimal data preprocessing (62).

Recently, Dawzynski *et al.* (63) reported that dietary PUFAs predominately increased several DHA-containing plasma PEs and plasmalogens following consumption of algal oil as a vegetarian marine source of n3-LCPUFAs in a small number of participants. In contrast, we found that most circulating classes of ionic lipids measured by MSI-NACE-MS, including omega-3 FA-containing PEs (e.g., PE 38:5, PE 38:6), LPEs (LPE 20:5, LPE 22:6), PIs (e.g., PI 40:6, PI 40:7), and LPCs (e.g., LPC 20:5, LPC 22:6) did not exhibit increases following high-dose FO supplementation with the exception of EPA and DHA as their NEFAs, but not docosapentaenoic acid (Fig. 1, supplemental Table S1, supplemental Fig. S1). These discordant results may be due to differences in marine supplement/composition (1.6 g/day DHA intake with unreported EPA content) and assay selectivity, as plasma lipidome changes were analyzed by direct infusion-MS/MS without chromatographic separation thereby being more prone to isobaric/isomeric interferences (63). Additionally, other phytochemicals and fat-soluble vitamin constituents present in algal oil may elicit distinct plasma lipidome changes in humans as compared to FO sources or purified DHA- or EPA-only supplements, including their predominate lipid form that impacts bioavailability (e.g., triglyceride vs. PL). Nevertheless, we replicated our findings in two independent trials that demonstrated that the sum concentration of PC 36:5 and PC 38:6 was most significantly correlated to the O3I as compared to other PC panels or a single PC species alone (Table 1, supplemental Table S3) when using a validated MSI-NACE-MS platform and a robust data workflow for credentialing ionic lipids (33). In our case, zwitter-ionic PCs were preferentially measured as their methylated cationic lipid derivatives with improved separation resolution and ionization response in MSI-NACE-MS under positive ion mode (34). Nevertheless, similar outcomes were measured for the same pair of underivatized PCs analyzed directly under negative ion mode (supplemental Table S1). Overall, lipidomic studies by MSI-NACE-MS demonstrated acceptable technical precision with a median CV = 13% as compared to the larger biological variance based on 44 circulating PCs consistently measured in most participants (supplemental Fig. S3). The two PC species identified as surrogate biomarkers of the O3I, namely PC 16:0_22:5 and PC 16:0_22:6, were characterized with high confidence by MS/MS after collision-induced dissociation experiments under positive and negative ion mode detection (supplemental Fig. S5). However, not all lipid species associated with FO, EPA, or DHA intake in our study comprised single resolved PC molecular species in MSI-NACE-MS, such as PC 38:5 that is comprised of two comigrating ions previously shown to be composed of PC 16:0_22:5 and PC 18:1_20:4 (34). This confounding effect may explain the poorer performance for certain PCs as putative O3I biomarkers (Table 1) when compared to fully resolved species in MSI-NACE-MS that lack isobaric/isomeric interferences. Nevertheless, independent replication using an orthogonal reversed-phase LC-MS/MS lipidomics method is warranted to further validate the findings in our study.

Among young, normal weight and otherwise healthy Canadian adults recruited in the placebo-controlled EPA and DHA-specific supplementation trial, their average O3I at baseline/placebo was (3.50 ± 0.68%) with most participants (74%) classified as having an O3I <4% (Fig. 5A). However, females had higher baseline O3I than males likely due to estrogenic effects that have been reported to upregulate DHA biosynthesis in women especially when taking oral contraceptives (64). In fact, most childbearing age and pregnant women do not meet their recommended dietary intake of omega-3 FAs, (65) which can increase the risk for premature and low-weight births (66). The O3I status in this cohort is considerably lower than a previous household survey of Canadian adults (20–79 years) reporting an average O3I of 4.5% with only 42% having <4% O3I (22), similar to data from a UK biobank study (27), and a dietary intervention involving company employees in Germany (67). All three studies reported a higher O3I status in women and older persons, including participants who regularly consumed fish and/or omega-3 FA supplements, but were not obese, and did not smoke tobacco. In fact, Stark *et al.* (58) reported that a suboptimal O3I status (<4% O3I) is prevalent in most global populations except for high consumers of seafood in Japan, Korea, Scandinavia, and certain indigenous groups not fully adapted to Western foods. Nevertheless, only modest increases in the O3I have been achieved by increasing the intake of omega-3 FA-rich seafood even in participants motivated to monitor their O3I status (67), with at least three fish servings per week plus dietary supplement use needed to achieve an O3I >8% that exceeds current guidelines by the American Heart Association (68). Our work confirmed that high-dose FO (3 g/day EPA + 2 g/day DHA) and EPA- or DHA-only supplements (3 g/day) significantly improved the O3I status in most study participants. However, there were considerable between-subject variations in treatment responses measured for circulating concentrations of PC 36:5 and PC 38:6 with a CV ranging from 55% to 73% for EPA- and DHA-only supplementation, respectively (Fig. 4). Overall, FO- and DHA-only supplements were most effective to increase O3I >8% (∼64-72%) in young Canadian adults as compared to EPA alone (∼11%) with DHA having a slightly greater impact in men than women reflecting their lower baseline O3I status (supplemental Table S4). These results are consistent with the greater potency and sex-dependence reported for DHA supplementation as compared to EPA (69). However, it is unclear how specific increases in the O3I that reflect changes in lipid membrane composition of erythrocytes are related to modulating long-term cardiovascular risk given the distinct mechanisms of action of EPA as compared to DHA in the body.

Overall, we demonstrated that serum concentrations for PC 36:5 and PC 38:6 had a better correlation with greater sensitivity to detect changes in O3I than the sum of DHA and EPA as their NEFAs (supplemental Fig. S4). Moreover, measured plasma concentrations for just these two circulating PCs retained most of their association with O3I, and only a marginal improvement was gained when reporting their fraction normalized to a total of 44 PCs (supplemental Fig. S8), which greatly simplifies and standardizes reporting. The differences in EPA and DHA activity for augmenting O3I status reflect the 50% higher dosage of EPA in FO as compared to DHA as well as the much lower content of EPA within erythrocyte membranes at baseline prior to supplementation (Fig. 3). This indicates that serum or plasma PC 36:5 may serve as a more sensitive blood biomarker for monitoring adherence to dietary/supplemental FO intake as well as an increasing number of EPA-specific therapeutic applications (50, 51, 52, 53). Indeed, recent studies have confirmed that the baseline O3I status, dose, and exact lipid formulation are primary factors that explain about 62% of the total variance in treatment responses to omega-3 FA supplements (70). In our study, treatment response variations were attributed mainly to biological sex as well as genotype differences that have been reported to effect fatty acyl desaturase and elongase activity, apolipoprotein E transport, and eicosanoid production (71). As expected, there were no changes in PC 36:5, PC 38:6, or their sum following intake of OO as placebo (Fig. 4C); however, modest increases were measured in linoleic acid containing PC 36:1 and an oleic acid containing PC 38:2 from baseline (supplemental Fig. S7). Overall, DHA supplementation elicited a 64% greater increase in the O3I from baseline as compared to EPA at the same dosage (Fig. 5A). Moreover, estimation of O3I status (62) from plasma PC 36:5 and PC 38:6 concentrations may be achieved by the use of EPA- and DHA-specific calibration curves (Fig. 5B). Recent studies highlight the distinctive effects that EPA-containing PCs have on membrane fluidity and structure than DHA alone, DHA/EPA mixtures, or omega-6-containing PCs, such as arachidonic acid (AA) (55). Indeed, Iwamatsu *et al.* (72) reported that the serum ratio of EPA to AA, but not DHA to AA, provided improved predictive accuracy as risk biomarkers of coronary artery disease, especially in patients with acute coronary syndrome.

Limitations of this study include the lack of selectivity for measuring neutral lipid classes (e.g., triglycerides, cholesteryl esters) by MSI-NACE-MS since it is optimal for the resolution and detection of various classes of anionic or zwitter-ionic (after methylation) lipids (34, 35). Previous ^13^C-labeled DHA single-dose feeding studies have demonstrated postprandial formation of PCs, PEs, CEs, and LPCs in plasma and subsequent uptake within erythrocytes plateaus between 9 and 72 h (73). However, our study focused on habitual/daily intake of high-dose omega-3 FA supplements after a minimum of 28 days while in a fasting state. Thus, we assessed long-term steady-state changes in lipidome profiles that primarily impacted specific PCs in circulation, and no changes were measured for other n3-LCPUFA-containing lipid classes (e.g., PEs, LPEs, LPCs, PIs) except for EPA and DHA as their NEFAs. Shorter sampling time points are needed to better assess the kinetics and uptake of specific yet resolvable PCs containing n3-LCPUFAs in circulation. As high-dose FO and EPA- or DHA-only supplements were comprised primarily as their triglycerides, future studies are needed to validate the generalizability of these two PCs as surrogate biomarkers of O3I after ingestion of other omega-3 FA formulations. Although previous large-scale epidemiological studies of omega-3 FAs from serum or plasma PLs have been reported, these procedures have relied on conventional FAME analysis by GC after fractionating lipid extracts by thin layer chromatography (74, 75). In contrast, our work offers a higher throughput approach for the direct analysis of intact yet specific PL species in blood specimens using MSI-NACE-MS or various LC-MS/MS lipidomic methodologies. Inadequate dietary intake of n3-LCPUFAs is not only relevant for high-risk adults prone to sudden cardiac death, depression, cognitive decline, and muscle loss with aging but also childhood health outcomes early in life (76).

## Conclusion

In summary, we explored the impact of high-dose n3-LCPUFA supplementation using FO-, EPA-, and DHA-specific formulations, on global changes in the blood lipidome profiles of healthy young adults. We first applied an accelerated data workflow when using MSI-NACE-MS to identify putative circulating lipid biomarkers associated with high-dose FO intake in a pooled subgroup analysis subsequently validated in two independent placebo-controlled trials. The sum concentration of only two circulating PCs, namely PC 16:0_20:5 and PC 16:0_22:6 in serum or plasma, provided the strongest correlation to the O3I that reflects local changes in erythrocyte membrane composition and cellular function after a minimum of 28 days of supplementation. However, PC 16:0_20:5 was more sensitive to omega-3 FA supplementation than PC 16:0_22:6 despite DHA intake generating greater increases in the O3I from baseline. Although MSI-NACE-MS was used for the discovery of circulating biomarkers of the O3I, other lipidomic platforms can also be used for their routine screening in small volumes of blood, including ion mobility-MS/MS and LC-MS/MS. The potential for noninvasive assessment of the O3I and its physiological effects following EPA and/or DHA supplementation in urine specimens may allow for more convenient nutritional screening applications. Future work will further validate these findings in a larger prospective cohort since circulating lipid pools of EPA and DHA are modifiable dietary risk factors correlated with longevity and vascular health. This work is critical to guide evidence-based dietary and lifestyle interventions for optimal health outcomes on an individual level.

## Data Availability

The data supporting this study are available in the article, the supplemental data, or from the corresponding author upon request.

## Supplemental data

This article contains supplemental data (32, 38).

## Conflict of interest

S. M. P. reports grants or research contracts from the US National Dairy Council, Canadian Institutes for Health Research, Dairy Farmers of Canada, Roquette Freres, Ontario Centre of Innovation, Nestle Health Sciences, Myos, National Science and Engineering Research Council, and the US NIH during the conduct of the study; personal fees from Nestle Health Sciences, nonfinancial support from Enhanced Recovery, outside the submitted work. S. M. P. has patents licensed to Exerkine but reports no financial gains from any patent or related work. All other authors declare that they have no known competing ﬁnancial interests or personal relationships that could have appeared to inﬂuence the work reported in this paper.
