# Supplementary material for: Lipidomic studies reveal two specific circulating phosphatidylcholines as surrogate biomarkers of the omega-3 index
Source: J Lipid Res. 2023 Sep 18;64(11):100445. doi: 10.1016/j.jlr.2023.100445 (PMC10622695; doi:10.1016/j.jlr.2023.100445)
Supplement: Supplemental Data [file mmc1.pdf]

SUPPLEMENTAL DATA

Lipidomic Studies Reveal Two Specific Circulating Phosphatidylcholines as Surrogate Biomarkers of the Omega-3 Index

Ritchie Ly[1], Brittany MacIntyre[2], Stuart M. Philips[3], Chris McGlory[3], David M. Mutch[2], Philip Britz-McKibbin[1*]

[1] *Department of Chemistry and Chemical Biology, McMaster University, Hamilton, Ontario Canada*

[2] *Department of Human Health and Nutritional Sciences, University of Guelph, Guelph, Ontario Canada.*

[3] *Department of Kinesiology, McMaster University, Hamilton, Ontario Canada*

Supplemental Tables S1-S4

Supplemental Figures S1–S8

Supplemental Table S1. Summary of 84 annotated serum PLs measured by MSI-NACE-MS when comparing high-dose FO/placebo post-treatment relative to baseline in pooled sub-groups.

Lipid Identification	*m/z*:RMT:mode[a]	Fold-change[b]	*p*-value[c]
FA 20:5	301.217:0.993:n	4.16	0.00133
FA 22:6	327.233:1.019:n	3.51	0.00112
PC 36:5	794.570:0.984:p	4.48	0.00599
PC 36:5*	838.560:0.704:n	1.67	0.0331
PC 38:5	822.601:0.992:p	1.49	0.0209
PC 38:5*	866.592:0.705:n	1.81	0.0267
PC 40:5*	894.623:0.711:n	2.05	0.0602
PC 40:6	848.616:1.002:p	1.33	0.0409
PC 40:6*	892.607:0.708:n	1.54	0.0258
PC 38:6	820.585:0.989:p	1.33	0.0482
PC 38.6*	864.576:0.707:n	1.54	0.0258
PC 40:8	838.632:0.994:p	1.60	0.0512
PC 39:7	832.585:0.994:p	1.48	0.0526
PC 35:4	782.606:0.983:p	0.64	0.0527
FA 16:1	253.217:0.993:n	0.68	0.0532
PC 34:3	770.570:0.982:p	0.63	0.0545
FA 20:3	305.249:0.972:n	0.60	0.0549
PC 36:3	798.601:0.987:p	0.46	0.0553
LPE 22:6	524.278:0.782:n	1.60	0.0581
PC 34:2	772.585:0.983:p	0.83	0.0584
FA 17:1	267.233:0.988:n	0.71	0.0589
PC 38:7	818.570:0.986:p	1.35	0.0592
PC O-42:5/P-42:4	864.684:1.016:p	0.77	0.0595
FA 18:2	279.233:0.981:n	0.67	0.0596
PC 37:4	810.601:0.988:p	0.75	0.0599
FA 20:1	309.280:0.966:n	0.64	0.0604
PC 36:4	796.585:0.985:p	0.77	0.0616
PI 40:6	909.550:0.992:n	2.21	0.0620
PC 40:4	852.648:1.005:p	0.78	0.0636
PI 40:7	907.534:0.996:n	0.61	0.0651
PC 38:4	824.616:0.994:p	0.74	0.0659
FA 18:3	277.217:0.982:n	0.80	0.0676
PC 39:4	836.616:1.004:p	1.36	0.0677
FA 18:0	283.264:0.978:n	0.82	0.0681
PC 36:6	792.554:0.983:p	1.39	0.0685
FA 16:0	255.233:0.993:n	0.72	0.0700
PC 35:3	784.622:0.985:p	0.71	0.0746
FA 12:0	199.170:1.027:n	0.86	0.0768
PC 33:3	756.554:0.978:p	0.73	0.0786
PC 38:3	826.632:0.999:p	0.65	0.0797
PC 35:5	780.590:0.979:p	0.78	0.0827
PC 40:5	850.632:1.004:p	1.15	0.0858

PC 37:5	808.585:0.987:p	0.86	0.0865
PC 37:3	812.616:0.993:p	0.76	0.0875
PC 30:0	720.554:0.973:p	1.47	0.0894
PC 37:6	806.570:0.986:p	1.26	0.0950
FA 17:0	269.249:0.982:n	0.85	0.107
FA 20:4	303.233:0.993:n	0.84	0.114
LPE 20:5	498.263:0.782:n	1.50	0.122
FA 14:1	225.186:1.009:n	0.79	0.123
PC 35:1	788.653:0.987:p	1.20	0.133
LPE 22:5	526.294:0.783:n	0.71	0.136
PC O-42:6/P-42:5	862.668:1.008:p	0.86	0.138
PC 33:0	762.601:0.984:p	1.28	0.140
FA 18:1	281.249:0.978:n	0.83	0.144
PC 36:2	800.616:0.992:p	0.92	0.144
PE 38:5	764.524:0.763:n	0.49	0.149
PC 34:1	774.601:0.985:p	0.90	0.153
PC 33:2	758.570:0.980:p	0.84	0.153
PC 31:1	732.554:0.975:p	1.20	0.184
PC 31:0	734.570:0.978:p	1.16	0.203
PC 34:4	768.554:0.978:p	0.76	0.204
PC 40:7	846.601:0.998:p	0.92	0.215
LPC 22:6	626.346:0.701:n	0.89	0.219
PC 32:0	748.585:0.980:p	1.14	0.234
PC 35:2	786.637:0.987:p	0.94	0.239
PE 37:6	748.492:0.765:n	0.58	0.291
FA 14:0	227.202:1.005:n	0.60	0.296
FA 15:0	241.217:0.999:n	0.85	0.296
PC 32:2	744.554:0.976:p	0.93	0.314
FA 22:5	329.249:0.97:n	0.57	0.320
PC 39:5	834.601:1.000:p	1.12	0.324
PE 39:7	774.508:0.766:n	1.65	0.330
FA 22:4	331.264:0.968:n	0.57	0.335
PC 32:1	746.570:0.978:p	0.97	0.399
PC 34:0	776.616:0.985:p	0.98	0.413
PC 36:1	802.632:0.990:p	1.02	0.414
PC 33:1	760.585:0.983:p	0.98	0.445
PC 36:0	804.648:0.994:p	1.01	0.461
PE 38:6	762.508:0.764:n	0.92	0.593
PE 35:5	722.477:0.764:n	1.39	0.628
FA 20:2	307.264:0.967:n	0.93	0.643
LPC 20:5	630.331:0.703:n	1.07	0.726
PE 37:5	750.508:0.764:n	0.96	0.864

[a] *Serum lipid extracts were analyzed by MSI-NACE-MS following methylation under positive ion mode (PCs), or underivatized under negative ion mode conditions (FAs, LPCs, PEs, LPEs, PIs). Top-ranked PCs (*) were also replicated in negative ion mode.*
[b] *Average fold-change in ion response for serum lipid following FO supplementation to baseline in pooled samples.*
[c] *Statistical significance of pooled serum lipid increase following FO supplementation using paired student's t-test.*

Supplemental Table S2. Clinical characteristics of participants in a placebo-controlled EPA or DHA (3.0 g/day) treatment intervention over a 90-day period.

		Clinical Characteristic			
**Grouping**	**Sex**	**Age**	**BMI**	**O3I (Baseline)**	**O3I (Post)**
Placebo (OO)	Female, *n* = 14	21 ± 1	24.6 ± 3.3	3.98 ± 0.73	3.76 ± 0.60
	Male, *n* = 13	21 ± 2	24.0 ± 4.0	3.46 ± 0.77	3.45 ± 0.59
EPA	Female, *n* = 14	21 ± 2	22.5 ± 2.8	3.61 ± 0.64	6.75 ± 1.20
	Male, *n* = 14	21 ± 2	23.4 ± 2.7	3.40 ± 0.87	6.23 ± 1.10
DHA	Female, *n* = 15	22 ± 2	22.7 ± 2.7	3.73 ± 0.51	8.04 ± 1.34
	Male, *n* = 13	23 ± 3	24.7 ± 3.8	3.15 ± 0.64	8.73 ± 0.97

Supplemental Table S3. Top-ranked plasma PCs and their panels that were associated with O3I following high-dose EPA or DHA supplementation as compared to OO as placebo. Plasma PC responses were reported using their relative peak areas.

Lipid Panel[a]	Number of Lipids (EPA, DHA)	Pearson Correlation (*r*)	*p*-value
PC 36:5 + PC 38:6[b]	2 (EPA: 1, DHA: 1)	0.764	3.0×10^{-33}
PC 36:5 + PC 38:5[c] + PC 38:6 + PC 40:6	3 (EPA: 1, DHA: 2)	0.711	5.6×10^{-27}
PC 36:5 + PC 38:5[c] + PC 38:6	2 (EPA: 1, DHA: 1)	0.706	1.7×10^{-26}
PC 38:6	1 (EPA: 0, DHA: 1)	0.663	1.5×10^{-22}
PC 36:5 + PC 38:5[c] + PC 40:5 + PC 36:6 + PC 38:6 + PC 40:6	5 (EPA: 2, DHA: 3)	0.636	2.5×10^{-20}
PC 36:5	1 (EPA: 1, DHA: 0)	0.494	1.2×10^{-11}
PC 38:5[c]	0 (EPA: 0, DHA: 0)	0.484	3.5×10^{-11}
PC 40:6	1 (EPA: 0, DHA: 1)	0.387	2.5×10^{-7}
PC 36:6	1 (EPA: 0, DHA: 1)	0.225	3.5×10^{-3}
PC 40:5	1 (EPA: 1, DHA: 0)	0.222	4.0×10^{-3}

[a] *All PCs are derivatized as their cationic phosphomethylesters to improve separation resolution and ionization responses in MSI-NACE-MS under positive ion mode detection.*

[b] *PC 36:5 = PC (16:0_20:5), PC 38:6 = PC (16:0_22:6).*

[c] *PC 38:5 was subsequently determined to be comprised of two unresolved lipid species, namely PC 16:0_22:5 and PC 18:1_20:4.*

Supplemental Table S4. Sex-dependence of EPA or DHA treatment intervention on changes in plasma PC 36:5 + PC 38:6 and erythrocyte PL membrane derived O3I.

Treatment	Sex	Age[a]	Δ(PC 36:5 + PC 38:6)[a]	ΔO3I[a]	ΔPC panel[b]	ΔO3I[c]
Placebo (OO)	F = 14	21.0 ± 1.4	0.43 ± 11.38	-0.22 ± 0.54	0.738	6.16×10^{-1}
	M = 13	20.9 ± 2.2	1.74 ± 8.51	-0.01 ± 0.65		
EPA	F = 14	21.0 ± 2.1	33.71 ± 16.15	3.14 ± 1.25	$\mathbf{1.89 \times 10^{-2}}$	5.71×10^{-1}
	M = 14	21.6 ± 2.0	19.30 ± 14.26	2.83 ± 1.14		
DHA	F = 15	21.5 ± 1.8	26.11 ± 16.15	4.31 ± 1.26	9.66×10^{-1}	$\mathbf{1.90 \times 10^{-2}}$
	M = 13	22.6 ± 2.8	25.80 ± 20.88	5.57 ± 1.10		

[a] *Reported values are mean ± standard deviation*
[b] *p-values calculated with Student's t-test after confirming normality using Shapiro-Wilk test ($p > 0.05$)*
[c] *p-values calculated with Mann-Whitney U test after confirming non-normality using Shapiro-Wilk test ($p < 0.05$)*

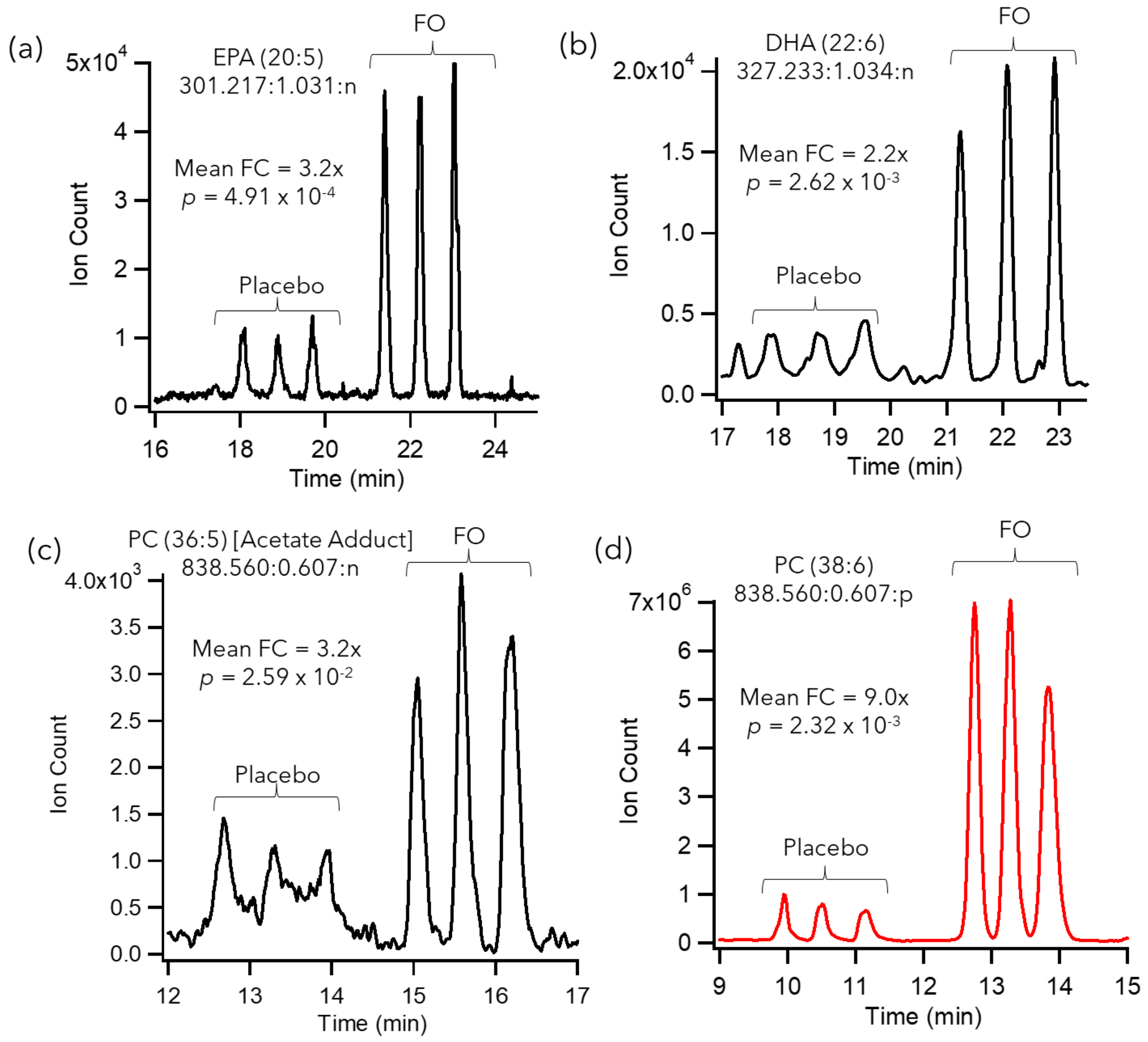

Supplemental Figure S1. Sub-group analysis of high-dose FO supplementation as compared to placebo/baseline showing response changes for **(a)** EPA and **(b)** DHA as their free NEFAs that were measured by MSI-NACE-MS under negative ion mode. **(c)** Independent replication of increase to PC 36:5 (underivatized and detected as its acetate adduct) by MSI-NACE-MS following FO ingestion under negative ion mode, which was sub-optimal for quantitative analysis given impact of matrix induced ion suppression and overall lower sensitivity under these operating conditions. **(d)** A DHA-containing PC species (PC 38:6) was confirmed to have a major increase in response following FO ingestion albeit to a lesser extent than PC 36:5 when using MSI-NACE-MS under positive ion mode (with FMOC/MTT labeling; refer to Figure 1b).

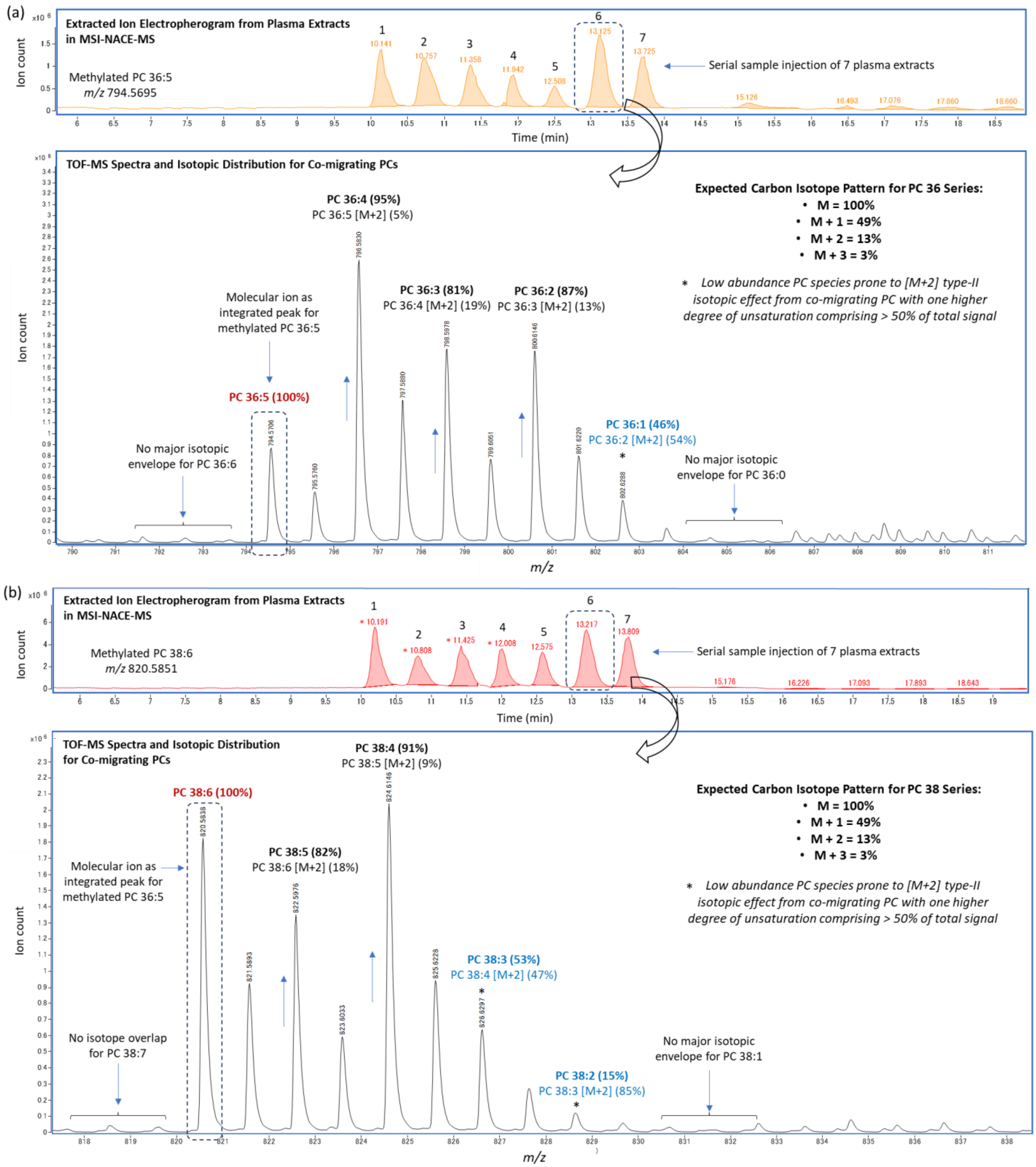

Supplemental Figure S2. Representative extracted ion electrophergrams for methylated **(a)** PC 36:5 and **(b)** PC 38:6 and their corresponding full-scan TOF-MS spectra highlighting potential type-II isotopic effects in MSI-NACE-MS when using a seven serial sample injection under positive ion mode. Both methylated PC 36:5 and PC 38:6 from plasma extracts did not have type-II isotopic effects due to the lack of co-migrating lipid isotopomers having one additional double bond (i.e., PC 36:6 or PC 38:7). In fact, the signal of the M+2 isomer for most other PC species are dominated by their co-migrating isotopomer having one additional double bond (> 80%) except for PC 36:1, and PC 38:3 and PC 38:2.

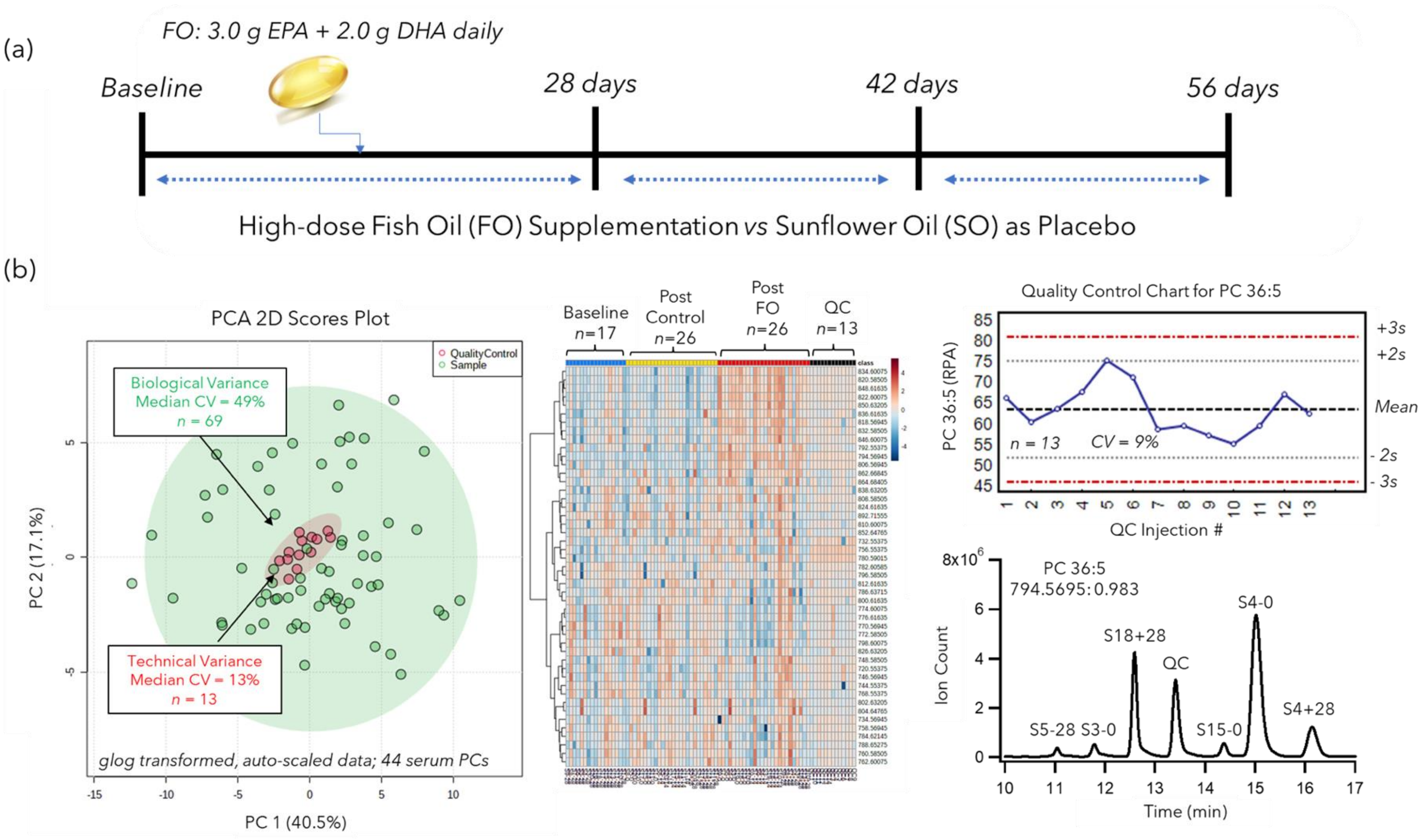

Supplemental Figure S3 (a) Study design overview of serum samples from a cohort of young women ingesting either sunflower oil (SO) or high-dose fish oil (FO) supplement over a 56-day period. **(b)** Summary of lipidomic data structure and data quality when using a 2D PCA scores plot and 2D hierarchical cluster analysis heatmap based on the analysis of 44 serum PCs (as their methylated phosphoesters), including putative O3I biomarkers identified after the sub-group analysis. The technical precision based on repeat analysis of pooled QC serum samples was acceptable (median CV = 13%, *n* = 13) when compared to the biological variability of the serum lipidome (median CV = 49%, *n* = 69) as also demonstrated in the control chart for PC 36:5. Extracted ion electropherogram depicts changes in the ion response for a randomized series of serum extracts analyzed by MSI-NACE-MS under positive ion mode conditions. Samples representing FO supplementation are noted by their large ion responses as compared to baseline or placebo samples, whereas the QC represents a pooled average response for the entire cohort.

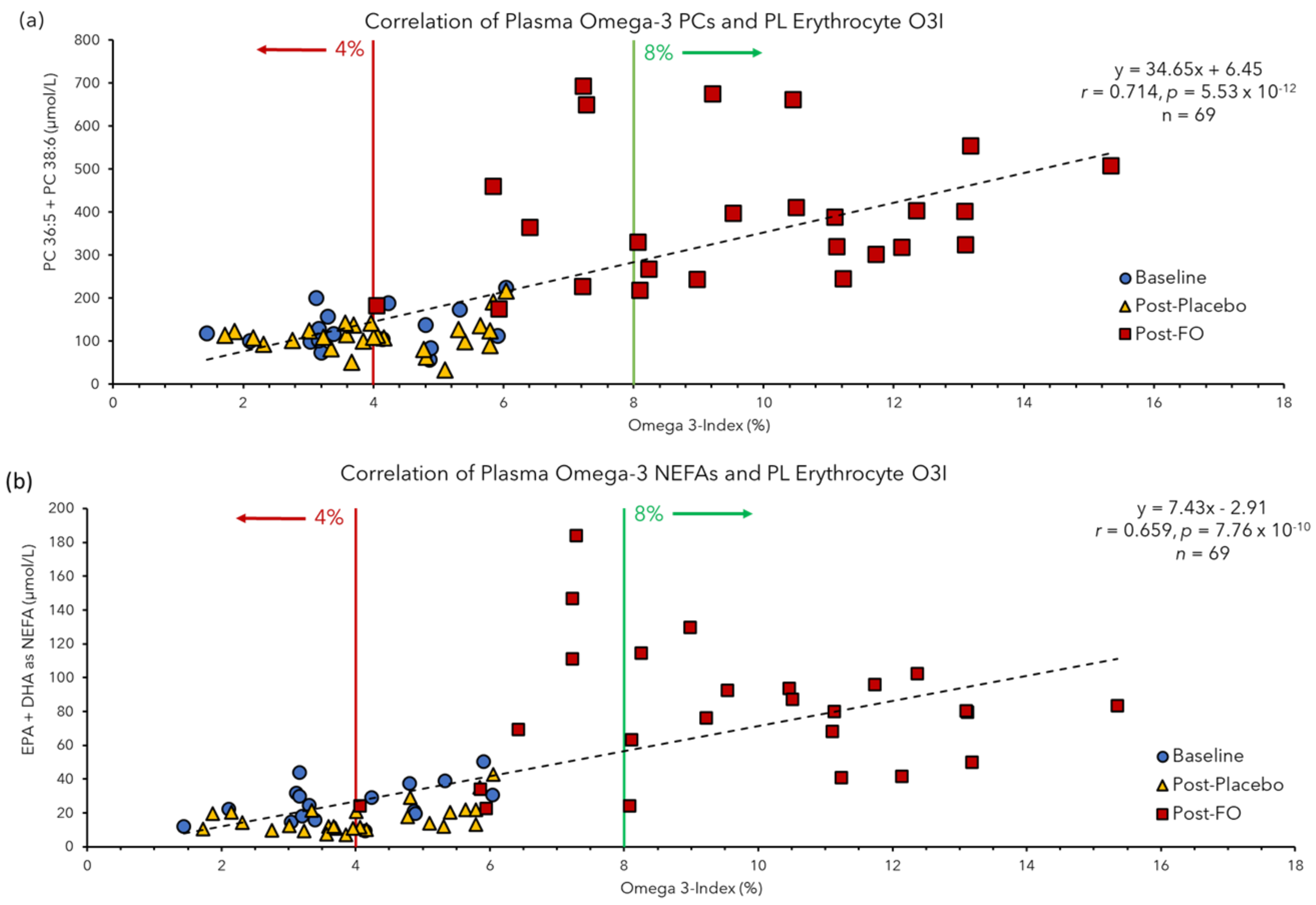

Supplemental Figure S4. A comparison of two circulating lipid pools in young adult women and their correlation with O3I following high-dose FO supplementation versus placebo (SO). Scatter plot for **(a)** serum ether extracts for the sum of PC 36:5 + PC 38:6 with O3I demonstrate a slightly stronger correlation to O3I with greater senstivity than **(b)** the sum of EPA+DHA as their NEFAs. NEFA data was previously reported in serum ether extracts using MSI-NACE-MS under negative ion mode conditions (Azab et al. *J. Lipid Res.* **2020**, 63: 933-944) .

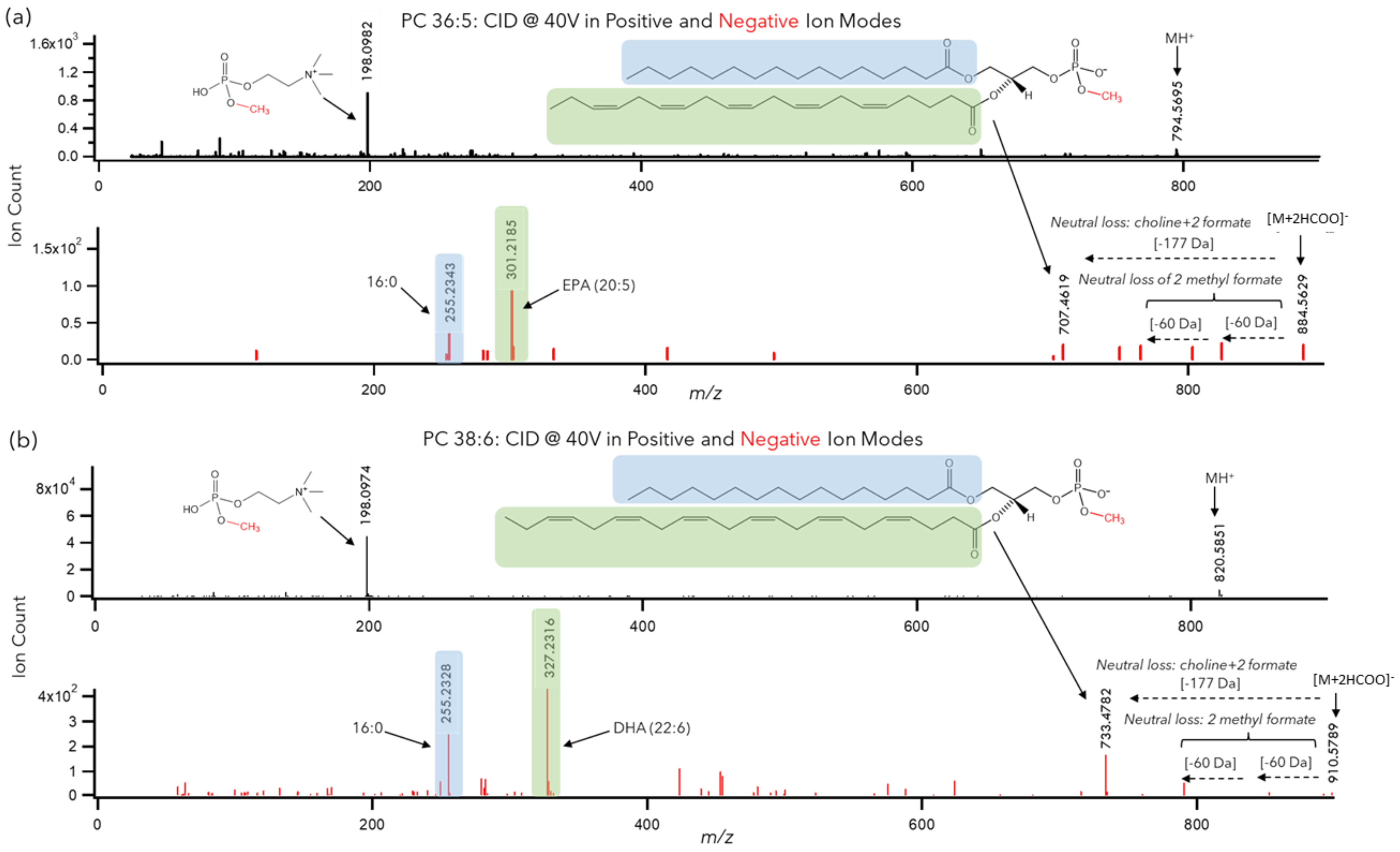

Supplemental Figure S5. Collision-induced dissociation MS/MS spectra acquired for methylated **(a)** PC 36:5 and **(b)** PC 38:6 from serum extracts under positive and negative ion mode using a Q-TOF under an optimal collision energy of 20 V. Annotation of MS/MS spectra confirm that a phosphatidyl choline head group likely containing palmitic acid (FA 16:0) and EPA (FA 20:5) or DHA (FA 22:6) in *sn-1* and *sn-2* positions, respectively based on their relative peak intensity (PC 16:0_20:5; PC 16:0_22:6). Chemical derivatization was performed using FMOC/MTT to render a positive charge on methylated PCs and improve their resolution and ion responses in MSI-NACE-MS under positive ion mode detection.

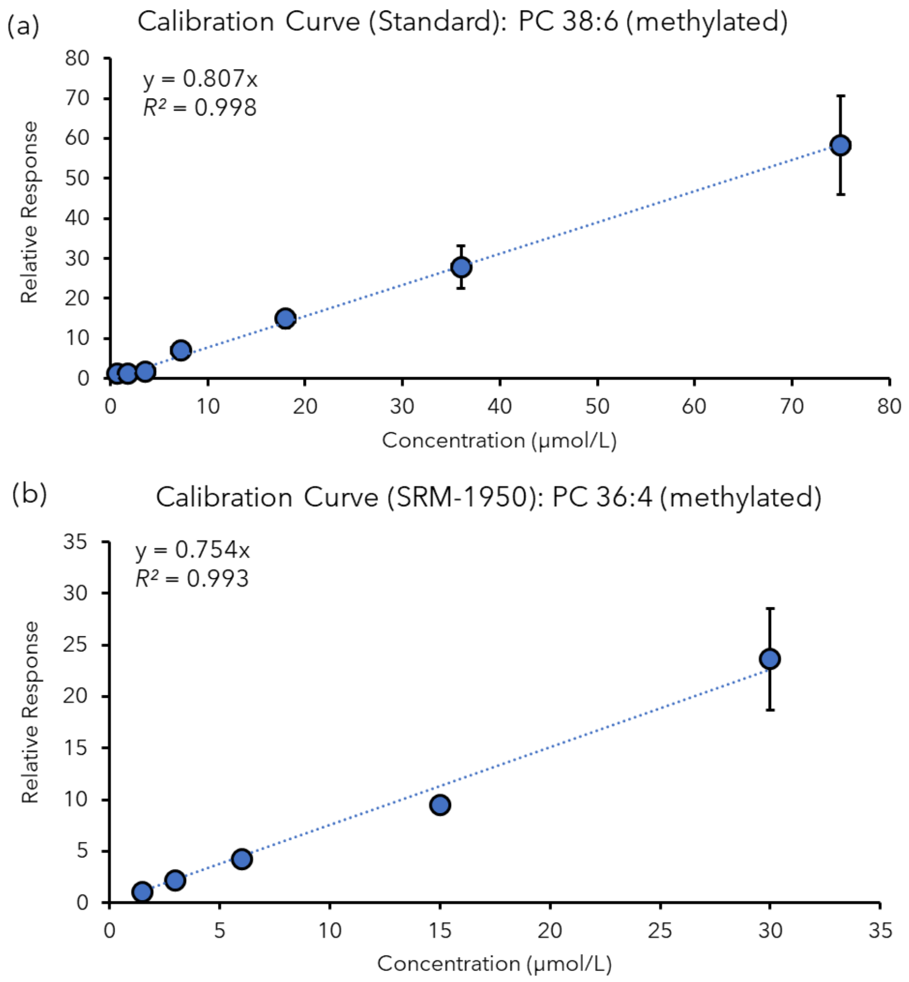

Supplemental Figure S6. Calibration curves used for (semi)-quantification of lead omega-3 containing PCs responsive to high-dose fish oil and EPA or DHA supplementation in human serum or plasma extracts when using pre-column methylation prior to MSI-NACE-MS analysis under positive ion mode conditions. Least-squares linear regression was performed with linearity over a 50 to 100-fold dynamic range when using **(a)** an authentic lipid standard for PC 38:6 (or PC 16:0_22:6), and **(b)** a surrogate lipid standard (PC 36:4) for PC 36:5 (or PC 16:0_20:5) based on serial dilution of NIST SRM-1950 (pooled human plasma) using consensus concentrations from lipidomics harmonization study (Bowden *et al. J. Lipid Res.* **2017** *58:*2275-2288). All PCs were derivatized using FMOC/MTT to generate cationic phosphomethylesters to improve their separation resolution and ionization response under positive ion mode with full-scan data acqusition, with normalization to a single internal standard, PC 32:0[D62].

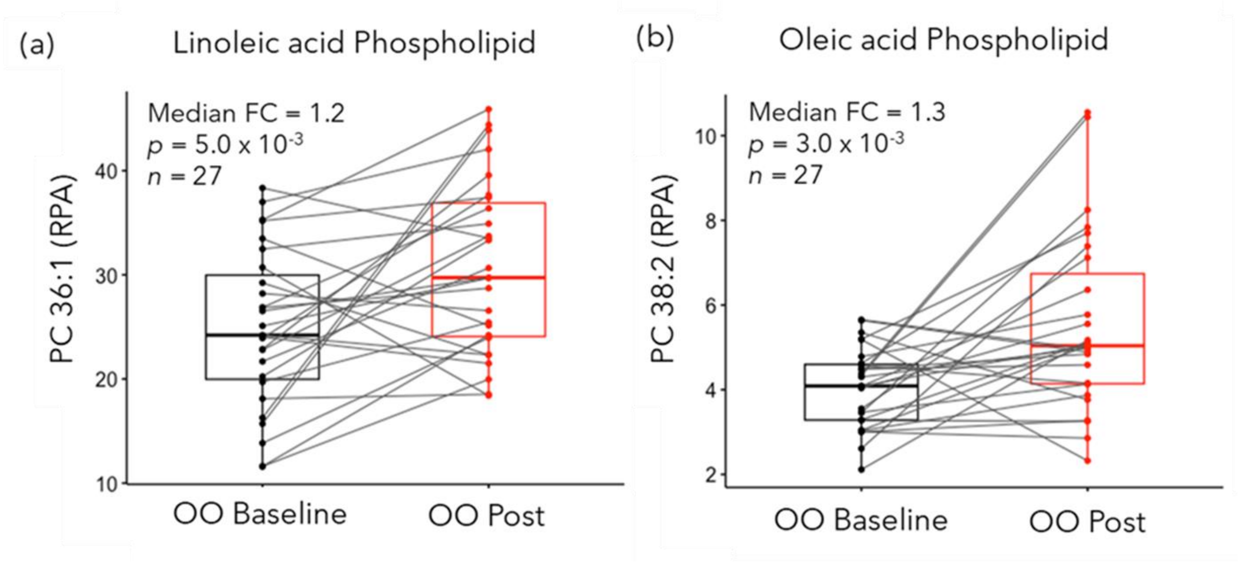

Supplemental Figure S7. Representative spaghetti plots shown for **(a)** linoleic acid and **(b)** oleic acid containing PCs (PC 36:1, PC 38:2) from plasma extracts that demonstrated only a modest increase from baseline in the olive oil (OO) placebo/control sub-group over 56 days. As expected, this control sub-group did not exhibit significant increases in circulating omega-3 containing PCs (PC 36:5, PC 38:6).

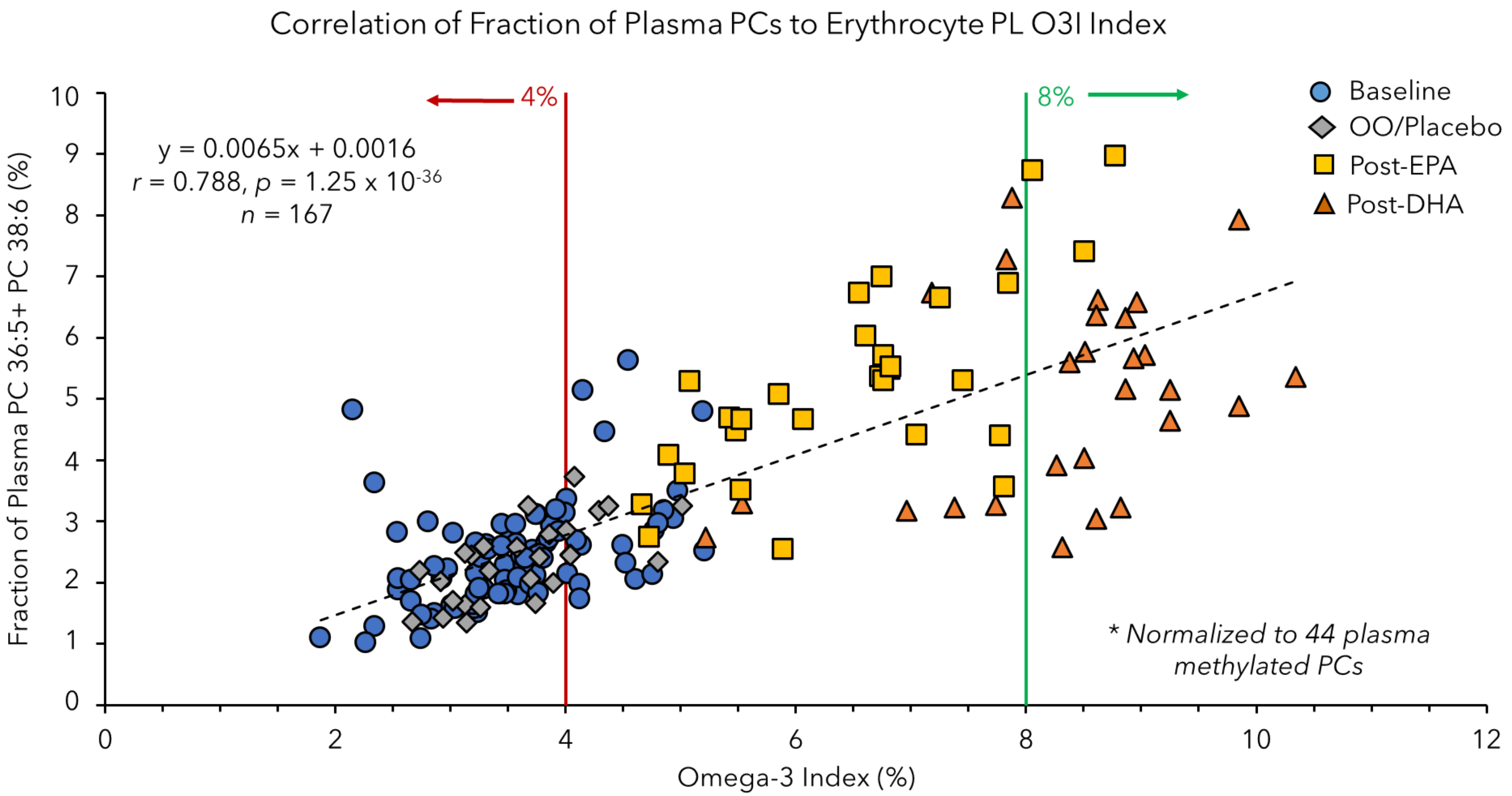

Supplemental Figure S8. A correlation plot highlighting the strong linear relationship between circulating plasma PC 36:5 + PC 38:6 expressed as a fraction of total PC (*n*=44) relative to erythrocyte PL membrane derived O3I measurements following high-dose EPA, DHA or olive oil (OO) supplementation as placebo from baseline in a cohort of young Canadian adults. Overall, the correlation strength to O3I was only modestly improved when using fraction (%) of PC 36:5 + PC 38:6 as compared to their absolute concentrations (μmol/L) in fasting human plasma. Plasma ether extracts were analyzed by MSI-NACE-MS under positive ion mode with full-scan data acquisition after lipid methylation using FMOC/MTT.
